# Emergent topological semimetal from quantum criticality

**DOI:** 10.1038/s41567-025-03135-w

**Published:** 2026-01-14

**Authors:** D. M. Kirschbaum, L. Chen, D. A. Zocco, H. Hu, F. Mazza, M. Karlich, M. Lužnik, D. H. Nguyen, J. Larrea Jiménez, A. M. Strydom, D. Adroja, X. Yan, A. Prokofiev, Q. Si, S. Paschen

**Affiliations:** 1 Institute of Solid State Physics, TU Wien, Vienna, Austria; 2 Department of Physics and Astronomy, Extreme Quantum Materials Alliance, Smalley-Curl Institute, Rice University, Houston, TX USA; 3 Laboratory for Quantum Matter under Extreme Conditions, Institute of Physics, University of São Paulo, São Paulo, Brazil; 4 Physics Department, Highly Correlated Matter Research Group, University of Johannesburg, Johannesburg, South Africa; 5 ISIS Neutron and Muon Source, Science and Technology Facilities Council, Rutherford Appleton Laboratory, Didcot, UK

**Keywords:** Phase transitions and critical phenomena, Topological matter

## Abstract

The electronic topology of a material is generally described by its Bloch states and the associated band structure, and can be altered by electron–electron interactions. In metallic systems, the interactions are usually treated through the concept of quasiparticles. Here we investigate what happens if no well-defined quasiparticles are present and show that a topological semimetal phase can emerge from the material’s quantum critical state. Using the non-centrosymmetric heavy-fermion compound CeRu_4_Sn_6_, which is intrinsically quantum critical, we show that the topological phase exhibits a dome structure as a function of the magnetic field and pressure. To understand these results, we study a Weyl–Kondo semimetal model at a Kondo destruction quantum critical point. Indeed, it exhibits features in the spectral function that can define topological crossings beyond the quasiparticle picture. Our results outline the importance of the interplay of quantum critical fluctuations and symmetry to search for other emergent topological phases.

## Abstract (web summary)

Examples of materials with non-trivial band topology in the presence of strong electron correlations are rare. Now it is shown that quantum fluctuations near a quantum phase transition can promote topological phases in a heavy-fermion compound.

## Main

Fundamental experimental^1–4^ and theoretical^5–8^ discoveries have led to the development of the flourishing field of topological quantum matter^9–13^. A large amount of (non-interacting) topological electronic materials has been predicted^14–17^, and for various materials, topological crossings have been directly visualized by angle-resolved photoemission spectroscopy^18,19^. Strongly correlated topological phases such as those identified in heavy-fermion systems represent a vast new opportunity, both in realizing novel topological phases of matter and in utilizing electron correlations to amplify the topological responses^20,21^. The identification of materials for such strongly correlated topology is inherently harder, given that the ab initio calculations of such materials remain a grand challenge and the best angle-resolved photoemission spectroscopy instruments still lack the energy resolution to image details within the extremely narrow heavy-fermion bands. One way to proceed is to combine symmetry constraints on the low-energy excitations of strongly correlated electrons with database surveys^22^. The present work suggests that there may be an alternative route, namely, that quantum phase transitions can nucleate emergent topological phases.

The most prominent example of an emergent phase in topologically trivial correlated materials is unconventional superconductivity that typically appears as a dome around a quantum critical point (QCP)^21,23,24^. ‘Optimum *T*_c_’, the highest superconducting transition temperature, is reached near the tuning parameter value at which non-Fermi liquid (NFL) behaviour emerges—frequently associated with strange metal characteristics. A thermodynamic argument for the formation of emergent phases is that the entropy accumulated at a QCP (for example, figure 3b of ref. ^21^) is released as a new phase forms. It is striking that this phenomenon is seen across many different materials classes, now even incorporating moiré heterostructures and other flat-band systems^20^. In most cases, the NFL behaviour extends to much larger temperatures and, at high temperatures, into a much wider tuning parameter range than the superconducting dome. As such, the ‘fan’ of NFL behaviour can be used as a pointer to the emergent phase.

Metallic heavy-fermion compounds are a well-established platform for realizing such QCPs and emergent superconductivity^21^. Kondo insulators—gapped representatives of heavy-fermion compounds—have larger energy scales than heavy-fermion metals, and larger control parameter changes are needed to tune them from one phase to another^25^. The intermediate situation—a semimetallic heavy-fermion state—is of great interest in the context of topological semimetals. We are not aware of any experiments that have investigated its potential connection to quantum criticality. Here we find that a Weyl–Kondo semimetal phase emerges from the quantum critical state of the heavy-fermion semimetal CeRu_4_Sn_6_, with a dome-like shape as a function of pressure and magnetic field. We propose that this situation may not be unique to CeRu_4_Sn_6_, and amounts to a new design principle for the discovery of other correlation-driven topological phases.

**Fig. 1:**
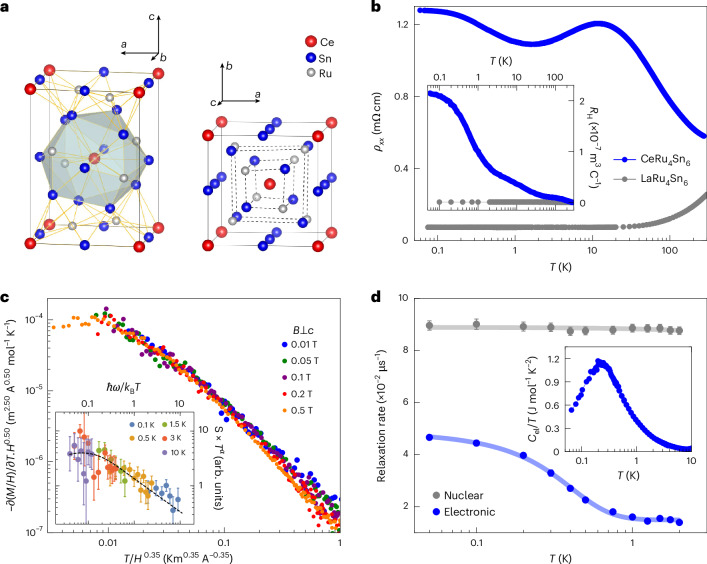
Overview and characterization of CeRu_4_Sn_6_. **a**, Unit cell (space group 121, $I\bar{4}2m$) viewed nearly along the tetragonal *b* (*a*) direction, with the polyhedron of nearest neighbours around the central Ce atom (left), and nearly along the *c* direction (right), where the broken inversion symmetry is evident. **b**, Temperature-dependent electrical resistivity (with *j*∥*c*) of CeRu_4_Sn_6_ and the non-magnetic reference compound LaRu_4_Sn_6_ in zero field (main panel), and Hall coefficient *R*_H_ of both compounds (inset). As expected for a semimetal, *R*_H_ of CeRu_4_Sn_6_ tends to saturate to a finite value at low temperatures. The characteristics of LaRu_4_Sn_6_ are typical of a simple metal. **c**, Signatures of pristine quantum criticality in the thermodynamic (main panel) and inelastic neutron scattering response (inset) of CeRu_4_Sn_6_. **d**, Temperature dependence of the electronic (blue) and nuclear contribution (grey) to the muon spin relaxation rate obtained from zero-field μSR measurements, indicating the absence of magnetic order (Supplementary Discussion 1). The error bars correspond to the standard error from the fits. For the electronic part, they are within the symbol size of the data points. Solid lines are guides to the eyes. The electronic specific heat coefficient *C*_el_/*T* increases smoothly with decreasing temperature (inset), as expected for quantum criticality; below 0.2 K, it decreases as the emergent Weyl–Kondo semimetal starts to develop. Panel **c** adapted with permission from ref. ^33^, AAAS.

CeRu_4_Sn_6_ crystallizes in the non-centrosymmetric tetragonal YRu_4_Sn_6_-type structure of space group 121 ($I\bar{4}2m$)^26^ (Fig. 1a). Together with the heavy constituting elements (atomic masses between 100 and 140) and a corresponding large atomic spin–orbit coupling, this may promote topological band crossings. Indeed, within the density functional theory plus Gutzwiller scheme, Weyl nodes were predicted to exist near the Fermi energy^27^. The temperature-dependent electrical resistivity *ρ*(*T*) of CeRu_4_Sn_6_ is typical of a semimetal, with a modest increase in *ρ* with decreasing temperature, whereas the non-*f* reference compound LaRu_4_Sn_6_ is a simple metal (Fig. 1b). This indicates that the Kondo interaction initiates the opening of a (pseudo)gap, although a full gap does not form (Supplementary Discussion 4). The tendency of the low-temperature Hall coefficient *R*_H_ to saturate to a small but finite value (corresponding to an effective carrier concentration of 0.014 per unit cell in a single-band picture; Fig. 1b (inset)) supports the classification of CeRu_4_Sn_6_ as a semimetal. In the first established Weyl–Kondo semimetal Ce_3_Bi_4_Pd_3_ (refs. ^28^^,29^), it was indeed concluded that Weyl nodes, situated within an otherwise Kondo insulating background^30^, prevent a full gap from opening (Supplementary Discussion 7). This feature also appears in Weyl–Kondo semimetal models^31,32^.

However, a previous magnetization and inelastic neutron scattering study of CeRu_4_Sn_6_ (ref. ^33^) revealed scaling collapses of the data (Fig. 1c), evidencing genuine quantum criticality of beyond Landau’s order-parameter-fluctuation type, as expected for a Kondo destruction QCP^34^, thereby seemingly excluding the presence of a topological phase. The scenario of quantum criticality is also consistent with the depolarization rate in zero-field muon spin rotation (μSR) and specific heat data (Fig. 1d) above 0.2 K: both increase smoothly with decreasing temperature, evidencing enhanced fluctuations, and lack signatures of ordering (Supplementary Discussion 1). All these set the stage for what we report next, namely, our discovery of Weyl–Kondo semimetal signatures that emerge out of this quantum critical state, showing that the two phenomena are, in fact, compatible with each other.

**Fig. 2:**
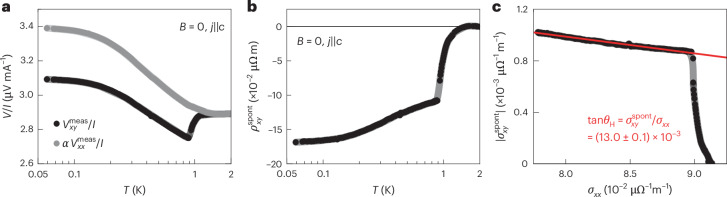
Spontaneous Hall effect in CeRu_4_Sn_6_. **a**, Temperature-dependent voltage measured on the Hall contacts ${V}_{xy}^{{\rm{meas}}}$ (black) and on the resistivity contacts ${V}_{xx}^{{\rm{meas}}}$ (grey), both divided by current *I*, with the latter scaled by the factor of *α* = 0.134 to ${V}_{xy}^{{\rm{meas}}}$ above the onset of the anomaly, all in zero magnetic field. Note that on this scale no anomaly can be discerned in ${V}_{xx}^{{\rm{meas}}}$ (Supplementary Discussion 8), indicating that it is a transverse response. The curves were measured at ambient pressure (1 bar) and are an example for the analogue behaviour observed at higher pressures. **b**, Zero-field Hall resistivity as a function of temperature, indicating the onset of a spontaneous Hall effect below 1 K. The curve was obtained from the difference ${R}_{xy}={R}_{xy}^{{\rm{meas}}}-\alpha {R}_{xx}$ (Supplementary Discussion 2). **c**, Absolute value of the spontaneous Hall conductivity ${\sigma }_{xy}^{{\rm{spont}}}$ as a function of the longitudinal conductivity *σ*_*xx*_, with temperature *T* as the implicit variable. Below 0.9 K, ${\sigma }_{xy}^{{\rm{spont}}}$ is linear in *σ*_*xx*_. The red line corresponds to a linear fit in this range; it provides an estimate of the Hall angle.

A transverse voltage contribution appears in CeRu_4_Sn_6_ below 1 K, in the presence of a longitudinal electric field but in the absence of any applied magnetic field (Fig. 2a). As CeRu_4_Sn_6_ is non-magnetic, this signal is identified as the spontaneous (nonlinear) Hall effect theoretically expected for a time-reversal-invariant but inversion-symmetry-broken Weyl semimetal^35^ and first observed in Ce_3_Bi_4_Pd_3_ (ref. ^29^). The corresponding temperature-dependent spontaneous (nonlinear) Hall resistivity ${\rho }_{xy}^{{\rm{spont}}}(T)$ is shown in Fig. 2b (Supplementary Discussion 2 provides details on contact misalignment corrections, reproducibility, resistivity anisotropy and normal magnetotransport effects, Supplementary Discussion 8 discusses spurious effects in the longitudinal channel, Supplementary Discussion 9 shows the nonlinear *V*(*I*) curves and Supplementary Discussion 10 addresses hypothetical inclusions). Plotting the spontaneous (nonlinear) Hall conductivity ${\sigma }_{xy}^{{\rm{spont}}}$ versus the longitudinal conductivity *σ*_*xx*_, with temperature as an implicit parameter, reveals a linear relationship below 1 K. This points to the intrinsic nature of the Hall response^36^. The slope ${\sigma }_{xy}^{{\rm{spont}}}/{\sigma }_{xx}\approx 13\times 1{0}^{-3}$, which corresponds to the Hall angle $\tan {\theta }_{{\rm{H}}}$, is around 100 times larger than the maximum value theoretically expected for TaAs (when the Fermi energy is artificially moved close to the energy of the Weyl nodes)^37^. Yet, it is distinctly smaller than the giant response found for Ce_3_Bi_4_Pd_3_ (ref. ^29^). One might be tempted to conclude that quantum criticality weakens the Weyl–Kondo semimetal state and that the two phenomena compete. Our measurements under pressure and magnetic field reveal that this is not the case. Instead, the phase discovered here is a new ‘emergent’ Weyl–Kondo semimetal, stabilized by quantum critical fluctuations.

**Fig. 3:**
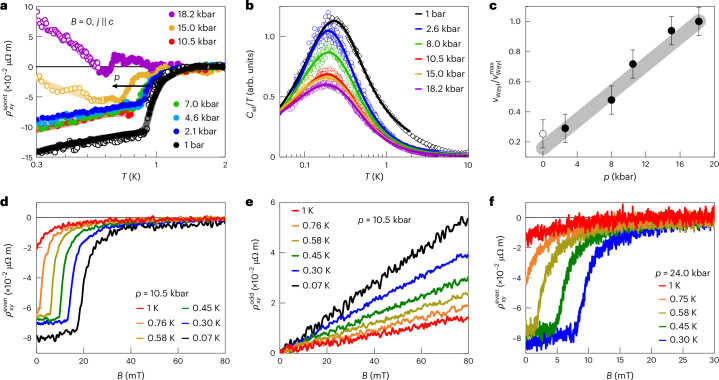
Pressure and magnetic field tuning of CeRu_4_Sn_6_. **a**, Spontaneous Hall resistivity ${\rho }_{xy}^{{\rm{spont}}}$ as a function of temperature for different pressures *p*, showing the suppression of the onset temperature *T*_H_ with increasing *p*. The 1-bar curve was measured after recontacting the sample (Supplementary Discussion 2), once with the same setup as the measurements under pressure (open symbols) and once using low-temperature transformers for higher resolution (full symbols). Above 15 kbar, the background subtraction via *αR*_*xx*_ introduces a sizable error due to the steep increase in *R*_*xx*_(*T*) at the lowest temperatures (Supplementary Discussion 4; strongly affected parts of the curves are shown with open symbols). ${\sigma }_{xy}^{{\rm{spont}}}(T)$ is less sensitive to the *R*_*xx*_ background and is almost fully suppressed at 18.2 kbar (Supplementary Discussion 2). **b**, Electronic specific heat coefficient *C*_el_/*T* versus *T*, scaled to the low-temperature ambient pressure data from Fig. 1d (black diamonds), for different pressures obtained via a.c. calorimetry (Supplementary Discussion 3 provides details on the measurement technique and the determination of the phonon contribution). The solid lines are phenomenological fits describing a crossover between the NFL behaviour and a contribution from linearly dispersing Weyl bands (Supplementary Discussion 3). Note that this ‘anomaly’ cannot be attributed to a nuclear Schottky contribution (Supplementary Discussion 3). **c**, Pressure-dependent Weyl velocity *v*_Weyl_ normalized to its maximum value ${\nu}_{{\rm{Weyl}}}^{{\rm{max}}}$. It was calculated from the Weyl contribution obtained from the fits in **b** (Supplementary Discussion 3). The increase in *v*_Weyl_ and the overall suppression of *C*_el_ with pressure indicate that pressure tunes the system away from the QCP. The error bars represent the largest standard error obtained from the fits of the different isobars. The grey line is a guide for the eyes. **d**,**e**, As expected for the finite-field extension of the spontaneous Hall effect, the magnetic-field-dependent Hall resistivity *ρ*_*xy*_(*B*) can be split into an even-in-field component ${\rho }_{xy}^{{\rm{even}}}$ (**d**) and an odd-in-field component ${\rho }_{xy}^{{\rm{odd}}}$ (**e**) (Supplementary Discussion 2). ${\rho }_{xy}^{{\rm{even}}}$ is suppressed with increasing *T* and *B*. **f**, The even-in-field Hall component ${\rho }_{xy}^{{\rm{even}}}(B)$ persists even up to the highest pressure of 24 kbar reached in these experiments, where the detection of the zero-field spontaneous Hall effect was no longer possible due to the rapid increase in *ρ*_*xx*_(*T*) in the relevant temperature range. Also here, the onset field ${B}_{{\rm{H}}}^{{\rm{even}}}$ is suppressed with increasing *p*, in agreement with the suppression of *T*_H_ presented in **a**.

We first discuss the measurements under hydrostatic pressure. We observe that both the magnitude of ${\rho }_{xy}^{{\rm{spont}}}$ and its onset temperature decrease with increasing pressure (Fig. 3a). The pressure-dependent specific heat data (Fig. 3b) suggest that pressure, just as magnetic field, suppresses quantum criticality, and thus, the spontaneous Hall signal is the strongest at ‘optimum’ quantum criticality. Note that no thermal phase transition appears under pressure tuning, leading one to speculate that order (likely of antiferromagnetic nature) may be stabilized by negative pressure (Supplementary Discussion 3). In an attempt to disentangle the Weyl–Kondo semimetal and quantum criticality contributions to the specific heat, we fit the data with a phenomenological function that describes a crossover between the two contributions (Supplementary Discussion 3). The lower border of the quantum critical fan estimated from this analysis increases with increasing pressure (Supplementary Fig. S14d), providing evidence that pressure drives the system away from quantum criticality. Also, the Weyl velocity *v*_Weyl_, which is related to the prefactor *Γ* of the Weyl–Kondo semimetal contribution *C*_el_ = *Γ* *T*^3^ (refs. ^31,28^), was estimated from this analysis and found to increase with increasing pressure (Fig. 3c). As *v*_Weyl_ is the slope of the Weyl dispersion, the smallest value corresponds to the flattest Weyl band and, thus, to the most strongly correlated state. Maximal correlation strength at quantum criticality is well established for topologically trivial heavy-fermion compounds and related materials^20,21^, but has not been demonstrated for correlation-driven topological semimetals.

Next, we describe our magnetic-field-tuning experiments. As shown previously^33^, the quantum criticality of CeRu_4_Sn_6_ is suppressed by magnetic fields^33^ (Fig. 1c). The Berry curvature probing quantity in finite fields is the even-in-field (nonlinear) Hall resistivity ${\rho }_{xy}^{{\rm{even}}}$ (ref. ^29^; Supplementary Discussion 2). Starting in the Weyl–Kondo semimetal phase, a finite magnetic field suppresses this even-in-field contribution (Fig. 3d,f shows example isobars), whereas the (normal) odd-in-field Hall resistivity ${\rho }_{xy}^{{\rm{odd}}}$ is featureless (Fig. 3e). Note that only tiny fields are needed to suppress the Weyl response, in contrast to the sizable fields needed for Weyl node annihilation in Ce_3_Bi_4_Pd_3_ (ref. ^30^) under Zeeman coupling tuning^38^. This confirms the result from pressure tuning that the Weyl–Kondo semimetal is the strongest under ambient conditions (*p* = *B* = 0), where it is quantum critical.

**Fig. 4:**
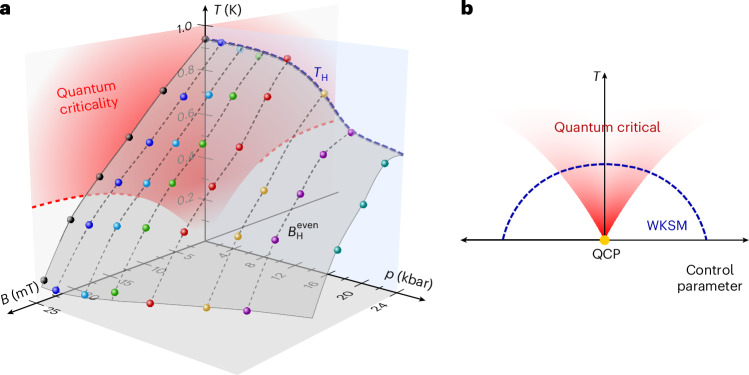
Pressure- and magnetic-field-tuned phase diagram of CeRu_4_Sn_6_. **a**, Temperature–pressure–magnetic field phase diagram with the onset temperatures *T*_H_ (in the *B* = 0 plane) and fields ${B}_{\rm{H}}^{{\rm{even}}}$ of the spontaneous Hall effect. The points were obtained from the zero-field ${\rho }_{xy}^{{\rm{spont}}}(T)$ curves shown in Fig. 3a and the even-in-field contribution to the Hall isotherms shown in Fig. 3d,f (Supplementary Discussion 2). These signatures, associated with Weyl–Kondo physics, are suppressed with both pressure and magnetic field and, thus, form a dome of Weyl–Kondo semimetal behaviour centred around the QCP. The red shading of the quantum critical fan in the *p* = 0 plane reflects the temperature range in which the scaling collapse of the inelastic neutron scattering holds, and the dashed boundary was derived from the NFL behaviour in the magnetization *M*(*T*) curves at different fields^33^. **b**, Cartoon of the phase diagram expected for an emergent Weyl–Kondo semimetal (WKSM), created by the quantum criticality of the beyond order-parameter-fluctuation type.

By defining Weyl–Kondo semimetal onset temperatures *T*_H_ and fields ${B}_{{\rm{H}}}^{{\rm{even}}}$ (Supplementary Discussion 2), we can construct a temperature–pressure–magnetic field phase diagram of CeRu_4_Sn_6_ (Fig. 4a). It shows a dome of Weyl–Kondo semimetal behaviour centred around the QCP (Fig. 4b), indicating that quantum critical fluctuations stabilize the Weyl–Kondo semimetal as an emergent topological phase. The understanding of this experimental observation poses a challenge. The definition of band topology roots in the particle (or quasiparticle) description of electrons in solids, via density functional theory in the simplest case or through effective renormalized band descriptions. At a Kondo destruction QCP, as evidenced in CeRu_4_Sn_6_, quasiparticles are expected to be absent, with recent direct experimental support for one material hosting this type of QCP^39^. How can a quantum critical state that loses quasiparticles nucleate a topological semimetal?

Clearly, the loss of quasiparticles invalidates the standard definition of topology in terms of Bloch states and the associated Berry curvature. Instead, it has recently been shown that topological nodes can be formulated as crossings of the single-particle spectral functions^40^. The key to this formulation is that the eigenfunctions of the single-particle Green’s function in interacting systems form a representation of the space group, in parallel to what Bloch functions do in the absence of interactions. Even for single-particle excitations that break the quasiparticle description, symmetry constraints ensure that the single-particle spectral functions, which are eigenvalues of the Green’s function, cross at the symmetry-dictated wavevectors^40^. This crossing corresponds to a topological node: a frequency-dependent Berry curvature, defined in terms of the eigenfunctions of Green’s function, specifies that the Berry flux surrounding such a node is quantized^41^.

**Fig. 5:**
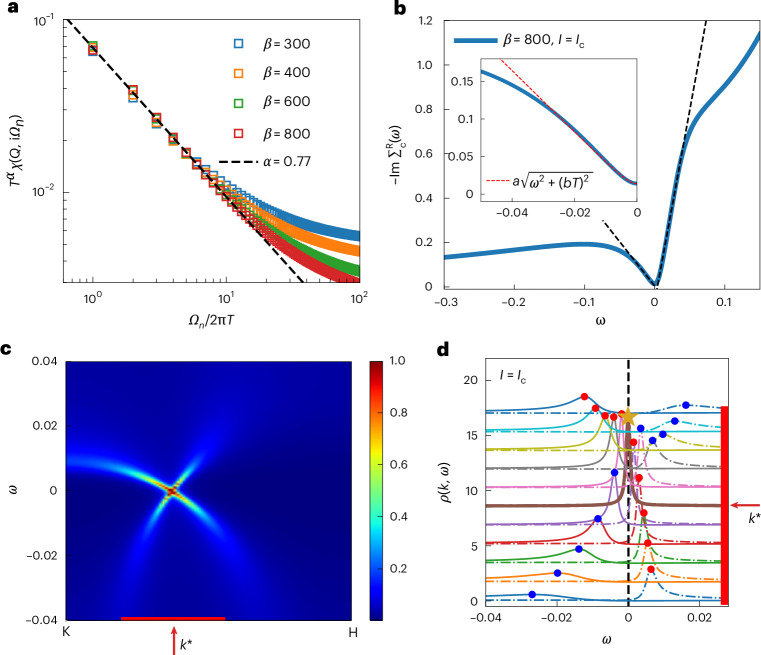
Kondo destruction quantum criticality nucleating a Weyl–Kondo semimetal. **a**, Kondo destruction QCP in a topological heavy-fermion model, defined by an Anderson-lattice Hamiltonian on a three-dimensional kagome lattice (Supplementary Discussion 5), as signified by the *ω*/*T* scaling of the dynamical lattice spin susceptibility. *β* is the inverse temperature. Throughout this figure, we use dimensionless quantities and set *ℏ* = *k*_B_ = 1. **b**, The imaginary part of the conduction electron (retarded) self-energy at the Kondo destruction QCP in the real frequency domain, obtained from a Padé decomposition, is linear in frequency in the low-frequency region (main plot). Taking into account the small but non-zero temperature *T*, it is well described by $-\Im{{\Sigma}}_{\rm{c}}(T,\omega)\approx a\sqrt{{\omega }^{2}+{(bT)}^{2}}$ at low frequencies, with the dimensionless fitting parameters *a* = 4.09 and *b* = 2.74 (inset). **c**, Spectral functions of the *f* electrons plotted along the high-symmetry K–H line of the Brillouin zone, where a spectral crossing is identified. **d**, Momentum-resolved energy distribution curves, with the red and blue dots highlighting the maxima of the corresponding dispersion. The red bars in **c** and **d** indicate the same range of wavevectors. At k*, the two branches overlap (maximum highlighted by the beige star), with the corresponding Weyl-nodal crossing of the spectral functions marked by the thick brown curve.

To demonstrate this effect near a QCP, we study a topological heavy-fermion model, corresponding to an Anderson-lattice Hamiltonian whose underlying (non-interacting) band structure contains symmetry-protected nodes (Supplementary Discussion 5). This model features a competition between the Ruderman–Kittel–Kasuya–Yosida and Kondo interactions, which we study in terms of an extended dynamical mean-field theory^42^. It realizes a Kondo destruction QCP^34^, where the Landau quasiparticles are destroyed. At this QCP, *k*_B_*T* emerges as the only energy scale in the system, giving rise to dynamical properties that obey a scaling in terms of the ratio of *ℏω* to *k*_B_*T*. We illustrate this by calculating the dynamical spin susceptibility at the wavevector where it peaks, finding it to have the dynamical scaling form with a fractional exponent (0.77; Fig. 5a). The quantum criticality is also captured by the single-particle excitations. Figure 5b shows the conduction electron self-energy in real frequency at the Kondo destruction QCP, obtained from an analytical continuation (Supplementary Discussion 5). In the relevant low-energy regime, the imaginary part of the retarded self-energy ($-\Im{{\Sigma}}_{\rm{c}}^{\rm{R}}(\omega)$) exhibits a linear-in-*ω* dependence, which implies that the quasiparticle residue *z* vanishes or, in other words, the loss of quasiparticles.

To see how this quantum criticality nucleates topology, Fig. 5c shows the spectral function of the *f* electrons along a high-symmetry K–H line in the Brillouin zone (Supplementary Discussion 5). Due to the symmetry constraint, the peaks of the spectral function cross, resulting in an NFL form of a Weyl point. We then examine the wavevector region marked by the red bar (Fig. 5c) and plot the momentum-resolved energy distribution curve (Fig. 5d). The red and blue dots track the peaks in the frequency dependence of the spectral functions, showing a Weyl crossing close to the Fermi energy. This provides the theoretical understanding of the emergent Weyl–Kondo semimetal and underpins the observations made in our experiments (for further aspects of theory–experiment agreement, see Supplementary Discussions 6 and 9).

Our work has advanced a new design principle for correlation-driven topological phases, namely, that a topological semimetal can emerge out of quantum criticality beyond the Landau order-parameter-fluctuation type. As there are established strategies to find such QCPs—particularly control parameter tuning^21^—we expect this insight to lead to the discovery of new emergent topological phases, perhaps with new signatures of topology and associated new functionalities. Will these phases be as abundant as (emergent) unconventional superconductivity? Both our experimental and theoretical results show that the signatures of quantum criticality persist into the emergent Weyl–Kondo semimetal phase. This differs from the situation of emergent superconductivity, where the entropy of the quantum critical fluctuations is released at the superconducting phase transition to bind the Cooper pairs, thereby restoring the quasiparticles of a (superconducting) Fermi liquid. Topological semimetals are not defined by a (Landau) order parameter but by topological indices and are, therefore, not delimited by a thermal phase transition. Consequently, there is no instantaneous entropy release and the Weyl–Kondo semimetal itself is expected to be an NFL. This could be tested by future experiments, for instance, by measuring whether shot noise is reduced from its Fermi-liquid value^39^. It would also be illuminating to examine whether the emergent topological semimetal is characterized by enhanced entanglement.

## Methods

### Synthesis

Single crystals of CeRu_4_Sn_6_ were grown using the floating-solution-zone travelling heater method in a mirror furnace, as described in detail in ref. ^43^. For more information on the crystal quality, see Supplementary Discussion 10. All samples used here were cut from the same large single crystal, which was oriented by Laue X-ray diffraction. As in the Laue patterns, the in-plane direction $c^{\prime}$ ([110]) of this tetragonal compound is barely distinguishable from the out-of-plane direction *c* ([001]), the identification of the $c^{\prime}$ and *c* directions was done via magnetization measurements, using the known magnetic anisotropy^44^.

### Measurement setups

Magnetotransport and specific heat measurements under hydrostatic pressure were performed using a piston cylinder cell from C&T Factory, with an inner diameter of 5 mm. Daphne 7373 oil was used as the pressure-transmitting medium, which is known to remain hydrostatic in the entire pressure range of our experiments^45^. Pressure was determined via the resistance of a HoCo_2_ manometer^46^, with an error of less than 5% determined from the width of the transition. The pressure cell was loaded with two CeRu_4_Sn_6_ single crystals. A Chromel/Au-0.07 at.% Fe thermocouple and three Au wires were attached to sample 1 for a.c. calorimetry measurements and four-point resistivity measurements. The Au-0.07 at.% Fe leg of the thermocouple also served as a current contact. In this case, current and magnetic field were applied along $c^{\prime}$ ([110]). Sample 2 was contacted in a conventional six-point configuration for resistivity and Hall effect measurements with current along *c* ([001]) and magnetic field along $c^{\prime}$. All electrical contacts to the samples were made by spot welding, with subsequent mechanical reinforcement using two-component silver epoxy (Polytec EC101), cured at 200 °C for 5 min. A detailed characterization of the a.c. calorimeter is given in Supplementary Discussion 3.

Measurements were carried out in an Oxford ^4^He flow cryostat (300 K to 2 K) and in an Oxford ^3^He/^4^He dilution refrigerator (2 K to 50 mK). Lock-in amplifiers (Stanford Research SR830, EG&G Signal Recovery Model 7260) and a.c. resistance bridges (Lake Shore LS370, Linear Research LR-700) were used for *R*_*xx*_ and *R*_*xy*_ measurements. A Stanford Research CS580 voltage-controlled current source provided the a.c. current for the lock-in measurements. Where needed, the measurement signals were preamplified (100×) at room temperature using two different types of preamplifier (Model 1900, EG&G Princeton Applied Research and SR554, Stanford Research Systems). For the high-resolution ambient pressure measurements in the dilution refrigerator, low-temperature transformers from CMR direct (100× gain) were installed at the still plate.

Specific heat under pressure was measured for sample 1 via the a.c. calorimetry technique^47,48^. The a.c. heating of the sample was generated via an a.c. current applied through two of the Au wires attached to the sample for transport measurements (Stanford Research CS580 voltage-controlled current source). The effective heater resistance *R*_h_ comprises the two contact resistances between the sample and the wires, as well as the sample resistance itself. The temperature dependence of *R*_h_ was determined experimentally for each pressure via a pseudo four-point measurement. The modulated sample temperature *T*_a.c._, which is approximately proportional to the inverse specific heat, was detected as a voltage with a custom-made Chromel/Au-0.07 at.% Fe thermocouple, preamplified 500 times at room temperature with a Stanford Research SR554 transformer preamplifier, read-out by a Stanford Research SR830 lock-in amplifier in the second-harmonic mode, and converted to temperature using generic thermopower curves from the literature^49,50^.

The heat capacity at ambient pressure was measured down to 0.4 K in a physical property measurement system from Quantum Design equipped with a ^3^He insert using the standard heat-pulse method. The sample was mounted on the platform using Apiezon N grease. Before the sample measurement, the empty platform was calibrated with N grease (addenda). Data at lower temperatures were measured on a custom-made setup in a closed-cycle ^3^He/^4^He dilution refrigerator from Leiden Cryogenics using a standard relaxation time technique. The sample platform consists of a silver frame and a silver sample stage suspended by superconducting NbTi wires, which also serve as electrical leads to the thermometer and heater (two different types of RuO_2_ chip resistor), thermally insulating them from the bath. The thermal link to the bath is provided via a bundle of Pt_0.9_Ir_0.1_ wires, fixed to the platform and the frame using silver epoxy. The sample was mounted on the platform with a small amount of silver paint (DuPont 4929N), which is expected to have a negligible contribution to the addenda. The empty platform was calibrated in a separate cooldown.

The zero-field μSR experiments were performed with the HiFi instrument at the pulsed muon facility ISIS of the Rutherford Appleton Laboratory, which provides a long data collection window (*t* > 15 μs). A single crystal of CeRu_4_Sn_6_ was mounted on a standardized silver sample holder, with the muon beam parallel to its *a* axis ([100]). It was measured between 2 K and 50 mK using a dilution refrigerator.

## Online content

Any methods, additional references, Nature Portfolio reporting summaries, source data, extended data, supplementary information, acknowledgements, peer review information; details of author contributions and competing interests; and statements of data and code availability are available at 10.1038/s41567-025-03135-w.

## Supplementary information

Supplementary Information Supplementary Discussions 1–10, Figs. 1–27 and Tables I and II.

## Data Availability

All data that are necessary to interpret, verify and extend this study are provided in the Article and are also available via Zenodo at 10.5281/zenodo.17475073 (ref. ^51^).
