# Supplementary material for: Emergent topological semimetal from quantum criticality
Source: Nat Phys. 2026 Jan 14;22(2):218–24. doi: 10.1038/s41567-025-03135-w (PMC12904783; doi:10.1038/s41567-025-03135-w)
Supplement: Supplementary file 1 — Supplementary Discussions 1–10, Figs. 1–27 and Tables I and II. [file 41567_2025_3135_MOESM1_ESM.pdf]

nature physics

Supplementary information https://doi.org/10.1038/s41567-025-03135-w

Emergent topological semimetal from quantum criticality

In the format provided by the authors and unedited

CONTENTS

Supplementary Discussion 1: Analysis of the muon spin rotation data 2
Supplementary Discussion 2: Analysis of the Hall effect data 4
Supplementary Discussion 3: Measurement and analysis of specific heat data 15
Supplementary Discussion 4: Pressure effect on the electrical resistivity 24
Supplementary Discussion 5: Details on the theoretical model and computations 25
Supplementary Discussion 6: Experiment vs theory 29
Supplementary Discussion 7: The Weyl-Kondo semimetal $Ce_3Bi_4Pd_3$ 31
Supplementary Discussion 8: Longitudinal resistance change near onset of the spontaneous Hall effect 32
Supplementary Discussion 9: Nonlinearity in the DC transport response 33
Supplementary Discussion 10: Single phase nature of $CeRu_4Sn_6$ samples 37

References 44

Supplementary Discussion 1: Analysis of the muon spin rotation data

In a muon spin rotation (μSR) experiment, a beam with polarized muons hits the sample (and the sample holder). Stopped muons gradually depolarize by the fields they experience at the stopping sites, and this depolarization is detected as an asymmetry signal. Zero-field (ZF) μSR is a sensitive probe of internal fields in the sample, both static (as in the case of magnetic order) and fluctuating. To extract information about the processes at play, ZF μSR spectra are generally fitted using the function

$$G_z(t) = A_0 \left[\frac{1}{3} + \frac{2}{3}(1-\sigma^2 t^2)\exp\left(-\frac{\sigma^2 t^2}{2}\right)\right] \exp\left[-(\lambda t)^\beta\right] + A_{\mathrm{BG}} \ , \quad (1)$$

where the sum in square brackets is the static Gaussian Kubo-Toyabe function describing a Gaussian distribution of local magnetic fields from nuclear spins at the muon stopping sites in the sample [1] and the stretched exponential decay $\exp[-(\lambda t)^\beta]$ is a general form to describe the contribution due to electronic moments in the sample. A_{BG} is the asymmetry contribution resulting from muons stopping in the silver sample holder, and is expected to be constant and temperature-independent. A_0 is the initial asymmetry from the sample, σ the nuclear, and λ the electronic depolarization or relaxation rate. Via the exponent β, different line shapes and thus electronic relaxation processes can be captured, most notably a Lorentzian line shape with $\beta = 1$ for dynamic relaxation arising from fluctuating electronic moments and a Gaussian line shape with $\beta = 2$ for static relaxation from static electronic moments.

Our μSR data on a single crystal of $CeRu_4Sn_6$ were taken between 2 K and 50 mK, with the muon beam parallel to the crystallographic a axis ([100]). Selected isotherms with fits described below are shown in Supplementary Fig. S1. As seen by eye, no sign of oscillations can be discerned from the spectra, and the initial ($t = 0$) asymmetry is temperature-independent, both providing evidence for the lack of magnetic order.

We now turn to a more quantitative analysis. Both Ru and Sn have isotopes with nuclear magnetic moments (^{99}Ru, abundance 13%, $-0.64\,\mu_{\mathrm{N}}$; ^{101}Ru, 17%, $-0.72\,\mu_{\mathrm{N}}$; ^{115}Sn, 0.34%, $-0.92\,\mu_{\mathrm{N}}$; ^{117}Sn, 7.7%, $-1.0\,\mu_{\mathrm{N}}$; ^{119}Sn, 8.6%, $-1.0\,\mu_{\mathrm{N}}$), leading to a sizable contribution to the ZF-μSR depolarization and a large (and almost temperature-independent) nuclear relaxation rate σ (Fig. 1d). By comparison, the nuclear moments of both Ag isotopes are small (^{107}Ag, 52%, $-0.11\,\mu_{\mathrm{N}}$; ^{109}Ag, 48%, $-0.13\,\mu_{\mathrm{N}}$), leading to a much smaller contribution. Ce has no nuclear moment but delivers the entire electronic moment.

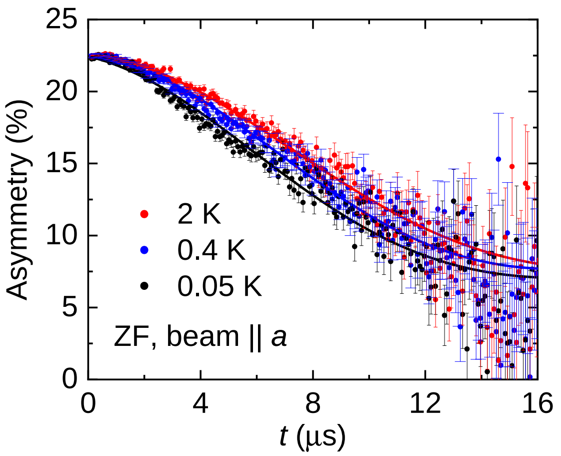

Fig. S1. **Temperature-dependent μSR spectra of $CeRu_4Sn_6$.** Zero-field (ZF) μSR spectra at 0.05 K, 0.4 K, and 2 K, with fits with $\beta = 1$ (solid lines) as described in the text. The error bars ΔA are calculated as $2/\sqrt{N_{\mathrm{total}}(t)}$, where $N_{\mathrm{total}}(t) = N_{\mathrm{forward}}(t) + N_{\mathrm{backward}}(t)$ is the sum in positron number on the forward and backward detector. Both the asymmetry A and its error bars ΔA are expressed as percentages.

The key question we address here is whether a quantitative fit will further underpin the nonmagnetic nature of $CeRu_4Sn_6$ down to the lowest temperature. We have thus fitted the 50 mK spectrum once with the exponent β fixed to 1, and once with β fixed to 2. The fit quality is much better for $\beta = 1$ (the standard deviation χ^2 is by 50% smaller than for $\beta = 2$). Indeed, if β is left open, the fit yields a β value close to 1. Also at 2 K, the fit with $\beta = 1$ is better than the one with $\beta = 2$ (χ^2 is smaller by 20%). These findings provide additional evidence for the paramagnetic nature of the material down to the lowest temperature. Note that this is different from the situation in $YbRh_2Si_2$, a strange metal in extreme proximity to a quantum critical point (situated at only 60 mT within the easy plane) but that ultimately orders antiferromagnetically below $T_{\mathrm{N}} = 70$ mK with tiny ordered moments. Despite its weakness, the order is unambiguously detected by the exponent β changing from about 1 above T_{N} to almost 2 below T_{N} [2].

To extract the temperature-dependent electronic relaxation rate $\lambda(T)$ (and the nuclear relaxation rate $\sigma(T)$ discussed already above) for all asymmetry isotherms, we thus fixed β to 1 (Supplementary Fig. S1). As shown in Fig. 1d, λ increases distinctly with decreasing temperature, indicating critical slowing down of the electronic relaxation. This is because due to the motional narrowing effect, the faster the magnetic fields fluctuate, the more slowly

the muons are depolarized.

Our conclusion of the absence of magnetic order in $CeRu_4Sn_6$ down to the lowest accessed temperature of 50 mK is in full agreement with a previous μSR study of polycrystalline $CeRu_4Sn_6$ [3].

Supplementary Discussion 2: Analysis of the Hall effect data

Hall contact misalignment correction. We denote the longitudinal electrical resistivity for an electrical current applied along the x direction as ρ_{xx}, the transverse electrical resistivity as ρ_{xy}. In our experiments, the samples were contacted by hand using the spot-welding technique (Methods) and small contact misalignments were unavoidable (Supplementary Fig. S2a). Thus, the voltage V_{xy}^{meas} measured on the Hall contacts (red) does not only contain the transverse voltage V_{xy} but also a misalignment contribution αV_{xx}. Likewise, the voltage measured on the electrical resistance contacts V_{xx}^{meas} contains the longitudinal voltage V_{xx} and a misalignment contribution βV_{xy}; the latter misalignment contribution might have been resolved as a tiny feature in high-resolution experiments, if not due to the Hall angle effect (Supplementary Discussion 8).

In standard Hall effect experiments, the misalignment contribution on the Hall contacts is eliminated by anti-symmetrizing the data taken in positive and negative magnetic fields via

$$V_{xy} = \frac{V_{xy}^{\mathrm{meas}}(+B) - V_{xy}^{\mathrm{meas}}(-B)}{2} \ . \tag{2}$$

The spontaneous (nonlinear) Hall response is measured without a magnetic field; thus, its analysis requires a different approach. The geometrical correction factor α is determined such that, at temperatures above the onset of the spontaneous (nonlinear) Hall response, $\alpha V_{xx}(T)$ and $V_{xy}^{\mathrm{meas}}(T)$ collapse (Fig. 2a; see also Supplementary Information of [4]). The genuine (purely transverse) Hall voltage is then calculated as

$$V_{xy}(T) = V_{xy}^{\mathrm{meas}}(T) - \alpha V_{xx}^{\mathrm{meas}}(T) \ . \tag{3}$$

This analysis was done for each isobar, and it was found that α increases slightly with pressure (Supplementary Fig. S2b). Unfortunately, when removing the sample from the pressure cell to perform the 1 bar measurements, contacts broke off, and the sample had to be re-contacted, resulting in a value of α different from the one obtained by linearly extrapolating

the plotted data in Supplementary Fig. S2b to $p = 0$. Note that the recontacting can also affect the size of the spontaneous Hall signal, which is expected to depend on the angle between the electrical field vector and the vector connecting a Weyl and the associated anti-Weyl node. We attribute the somewhat larger value of $\rho_{xy}^{\mathrm{spont}}$ at 1 bar in Fig. 3a to this effect. The onset temperatures T_{H} of the spontaneous (nonlinear) Hall effect, used to construct the phase diagram in Fig. 4a, were determined as the temperatures where the spontaneous (nonlinear) Hall resistivity $\rho_{xy}^{\mathrm{spont}}$ reaches 50% (error bars at 35% and 65%) of the crossover height (Supplementary Fig. S2c). At 15 kbar and above, where the spontaneous Hall conduc-

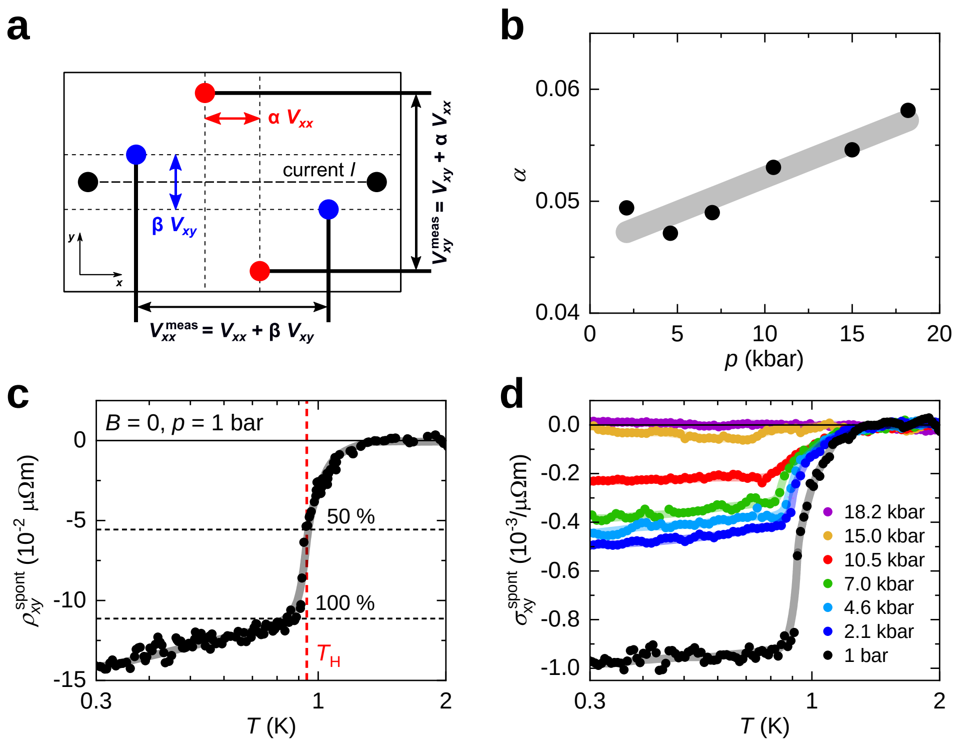

Fig. S2. **Analysis of spontaneous Hall data.** **a**, Cartoon of the misalignment contributions to the voltages measured on the resistivity (blue) and Hall effect contacts (red). **b**, Geometrical correction factor α for the sample in the pressure cell. The gray line is a guide to the eyes. **c**, Temperature-dependent spontaneous Hall resistivity $\rho_{xy}^{\mathrm{spont}}$ at ambient pressure after misalignment correction, with the definition of the onset temperature T_{H}. **d**, Spontaneous Hall conductivity $\sigma_{xy}^{\mathrm{spont}}$ as a function of temperature for different pressures. The 1 bar curve was taken after recontacting the sample, which was necessary because contacts broke off when removing it from the pressure cell (see text).

tivity is already strongly suppressed (Supplementary Fig. S2d) but $R_{xx}(T)$ increases steeply (Supplementary Discussion 4), minimal mistakes in α have sizable effects on $\rho_{xy}^{\text{spont}}(T)$ (open symbols in Fig. 3a); nevertheless, the onset temperature T_{H} can still be identified.

In finite magnetic fields, the Berry curvature-induced spontaneous Hall effect finds continuation in the form of an even-in-field contribution ρ_{xy}^{even} to the Hall resistivity (Fig. 3d,f). To extract it from the raw resistance data, the $R_{xy}^{\text{meas}}(B)$ traces at each T and p are first corrected for small B offsets (typically $< 5\,\text{Oe}$ when the magnet was carefully oscillated before the measurement) due to remanent magnetic fields. Then, the data are symmetrized via

$$R_{xy}^{\text{symm}} = \frac{R_{xy}^{\text{meas}}(+B) + R_{xy}^{\text{meas}}(-B)}{2} \,, \tag{4}$$

and subsequently corrected for a magnetoresistance contribution from contact misalignment (similar to Eqn. 3) via

$$R_{xy}^{\text{even}}(B) = R_{xy}^{\text{symm}}(B) - \alpha R_{xx}^{\text{symm}}(B) \,. \tag{5}$$

In this case, the scaling factor α is determined such that, at magnetic fields above the onset of the even component, $R_{xy}^{\text{symm}}(B)$ and $\alpha R_{xx}^{\text{symm}}(B)$ collapse.

This analysis was done for each isotherm at different pressures leaving α open. Similar to the zero-field case (Supplementary Fig. S2b), α was found to change slightly with pressure. In addition, a small change ($< 5\%$) of α with temperature is observed. The even component $R_{xy}^{\text{even}}(B)$ is detected up to the highest reached pressure of $p = 24\,\text{kbar}$, where detection of the zero-field response as a function of temperature has become too imprecise (see above). The onset fields $B_{\text{H}}^{\text{even}}$ of $R_{xy}^{\text{even}}(B)$, used to construct the phase diagram in Fig. 4a, were determined as the fields where $|R_{xy}^{\text{even}}|$ reaches 50% of its full height (similar procedure as in Supplementary Fig. S2c).

Reproducibility. As explained above, the transverse voltage contacts cannot be perfectly aligned and thus a misalignment correction is needed to extract the spontaneous Hall effect resistivity. Here we show that this procedure is well-controlled and leads to reproducible results. We have prepared a sample with 8 contacts (by adding two current contacts to the situation in Supplementary Fig. S2a, using the sample of Fig. 2), positioned roughly on two perpendicular lines, thus allowing us to measure the spontaneous Hall signal on essentially the same sample volume but along two approximately perpendicular directions. For both measurement configurations, the spontaneous Hall signal is quite similar (Sup-

plementary Fig. S3), even though the scaling factors needed to subtract the misalignment contribution are somewhat different (α = 0.134 and 0.186, respectively). This is precisely what is expected for the Berry-curvature-induced spontaneous Hall effect and shows the robustness of the contact misalignment correction procedure and also of the phenomenon as such.

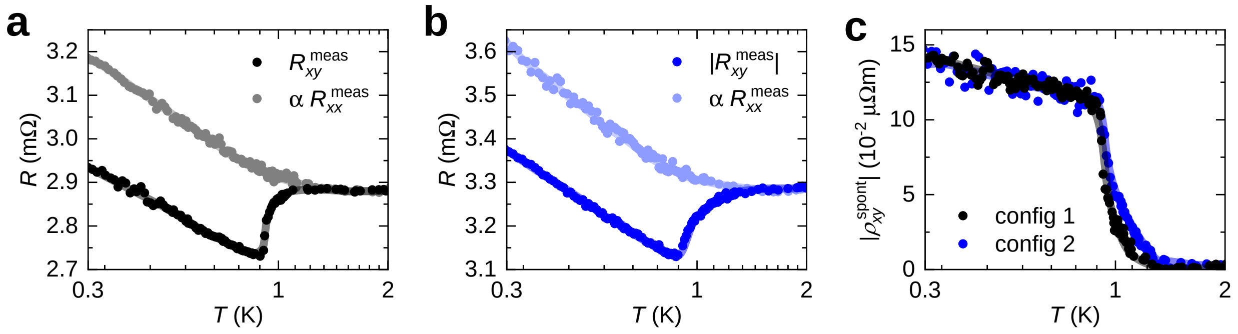

Fig. S3. **Spontaneous Hall effect measured by exchanging current and voltage probes.** **a**, Temperature-dependent resistance measured on the Hall contacts (black) and on the resistivity contacts (gray), with the latter scaled by the factor $\alpha = 0.134$. **b**, Same experiment as in **a** but with exchanged current and Hall contacts. In this case, the scaling factor was $\alpha = 0.186$. **c**, Absolute value of the spontaneous Hall resistivity $\rho_{xy}^{\mathrm{spont}}$ (after background subtraction) for the two modifications, showing that the signal is very similar.

Finally, we show that the spontaneous Hall resistivity is also reproducible from sample to sample (Supplementary Fig. S4). Note that the magnitude (and sign) of the spontaneous Hall signal is expected to depend on the angle between the electrical field vector and the vector connecting a Weyl and the corresponding anti-Weyl node. Thus, we show scaled absolute values here. The parameters used for the scaling are given in Table I.

To further demonstrate the robustness and reproducibility of our results and to support the hydrostatic pressure effect, we synthesized and investigated single crystals of $CeRu_4(Sn_{5.8}Ge_{0.2})$. This isoelectronic substitution (of Sn with Ge) reduces the lattice parameter, which corresponds to chemical pressure. As in pure $CeRu_4Sn_6$, an anomaly is observed in the Hall channel, but absent in the longitudinal channel, and is identified as a spontaneous Hall effect (Supplementary Fig. S5). Note that here the transverse signal shows a sizable *in*crease above the positive misalignment contribution ($\alpha\rho_{xx}^{\mathrm{meas}}$) making it highly unlikely that the Hall signal is due to a superconducting inclusion (Supplementary

TABLE I. Absolute values of the onset temperature T_{H} of the spontaneous Hall effect and magnitude of the spontaneous Hall resistivity signal $|\rho_{xy}^{\mathrm{spont}}|$ reached just below the initial strong increase, for the different measurements presented in Supplementary Fig. S4.

measurement	T_{H} (K)	$\|\rho_{xy}^{\mathrm{spont}}\|$ ($\mu\Omega$m)
sample #1, configuration 1	0.950	0.125
sample #1, configuration 2	0.995	0.126
sample #2, configuration 1	0.934	0.277
sample #2, configuration 2	0.961	0.442

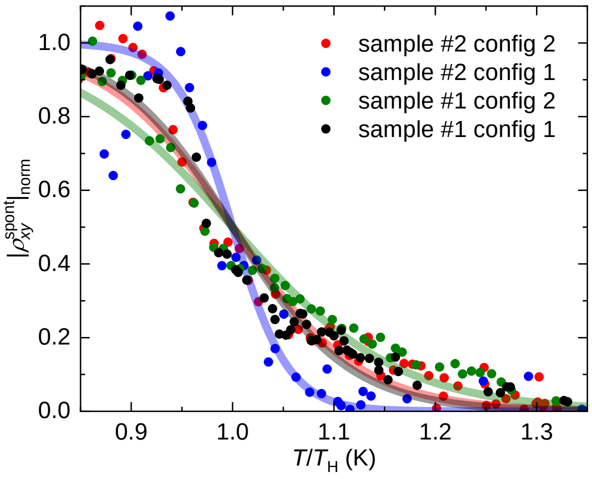

Fig. S4. **Reproducibility of the spontaneous Hall effect.** Absolute value of the spontaneous Hall resistivity $\rho_{xy}^{\mathrm{spont}}$ as a function of temperature for 4 different measurements on two different samples. Configuration 1 and 2 for each sample correspond to measurements with exchanged current and Hall contacts. All curves are normalized to the size of the anomaly and the temperature is scaled to the midpoint of the onset T_{H} for better comparability. Solid lines are guides to the eyes.

Discussion 10). In finite fields, it finds continuation as an even-in-field contribution, just as in $CeRu_4Sn_6$. The suppression of the onset temperature (to about 0.3 K) by chemical pressure is consistent with the trend observed in $CeRu_4Sn_6$ under hydrostatic pressure, which provides independent evidence for the pressure tuning effect.

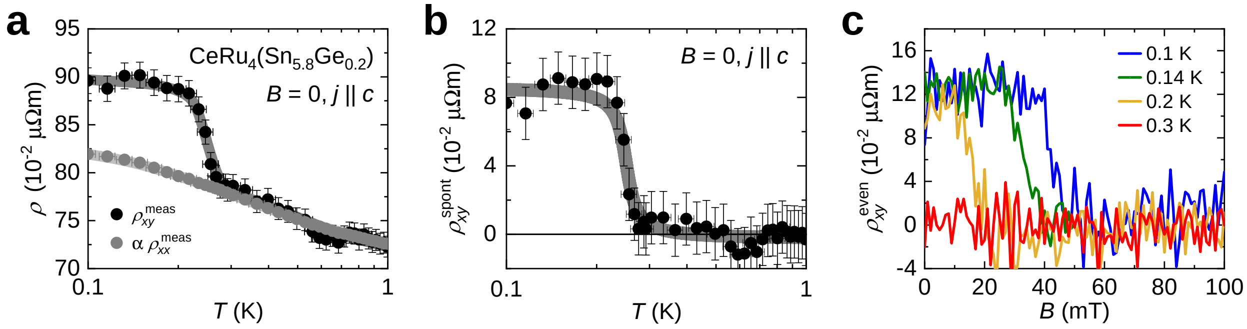

Fig. S5. **Spontaneous Hall effect in $\mathbf{CeRu_4(Sn_{5.8}Ge_{0.2})}$.** **a**, Temperature-dependent resistivity measured in zero magnetic field on the Hall contacts $\rho_{xy}^{\rm meas}$ (black) and the longitudinal contacts $\rho_{xx}^{\rm meas}$ (gray), with the latter scaled by a factor $\alpha = 0.04$, indicating the onset of a spontaneous Hall effect below 0.3 K. Note that the anomaly has the same sign as the ρ_{xx} background (both are positive), making it highly unlikely to result from a superconducting impurity. **b**, Spontaneous Hall resistivity as a function of temperature. The lower onset temperature compared to pure $CeRu_4Sn_6$ conforms to the expectation from pressure tuning, as the substitution of Sn with Ge corresponds to positive chemical pressure. **c**, The spontaneous Hall effect extends into an even-in-field Hall component $\rho_{xy}^{\rm even}(B)$ that is suppressed with increasing T and B, just as in $CeRu_4Sn_6$. The somewhat larger fields required to suppress the Hall signal are attributed to microscopic differences between chemical and hydrostatic pressure, such as increased disorder. The error bars in panel a and b correspond to the scattering of the raw (unaveraged) data. Solid lines are guides to the eyes.

Hall contribution from resistivity anisotropy. Here we show that the anisotropy of the electrical resistivity of $CeRu_4Sn_6$ [5] cannot create the observed spontaneous Hall effect. Within the tetragonal plane (between the [100] and [110] directions, called a and c', respectively), the electrical resistivity anisotropy is less than 5%, and could even be entirely due to (unavoidable) uncertainties in the determination of the form factor A/l ($\rho = R \cdot A/l$, where R is the measured resistance of a sample with cross-section A and length l between the voltage contacts in a 4-point measurement). The anisotropy between the tetragonal plane and the perpendicular direction ([001] or c) is about a factor of 2. To assess the effect this "trivial" (anisotropy as opposed to Berry curvature-related) contribution may have on the Hall angle and Hall conductivity, three different situations should be discriminated:

a. If the electrical current is applied along the principal directions of the tetragonal lattice, the anisotropy has no effect, regardless of which principal directions are combined.

b. If the current and Hall voltage contacts are positioned along different directions within the (essentially isotropic) tetragonal plane, no effect will result, regardless of the directions within this plane.

c. If the electrical current and Hall voltage contacts are positioned along arbitrary other directions, small trivial Hall contributions might occur. However, these should be present at all temperatures and not appear only as Kondo coherence sets in.

This can be seen by solving the Boltzmann equation in the presence of an electric field $\mathcal{E}$ but without Berry curvature term perturbatively (i.e., to the leading order), where the non-equilibrium distribution function f is given by $f_0 + f_1$, with the equilibrium (Fermi-Dirac) distribution f_0 and (with $\hbar$ set to 1)

$$f_1 = e\tau\mathcal{E}_a\partial_a f_0 \quad .$$

The repeated indices mean summation over. The anisotropic electrical current induced by the "normal" (i.e., not Berry curvature-related) velocity term is

$$j_a = -e\int_k \partial_a\epsilon_k f_1 \quad . \tag{6}$$

Combining these equations, we find the current induced by the normal velocity to be

$$j_a = -e^2\tau\int_k (\partial_a\epsilon_k)\,(\partial_\mu f_0)\,\mathcal{E}_\mu \quad . \tag{7}$$

An electric field applied along an arbitrary direction can be expressed as the combination of its components along the three principle axes, i.e., $\mathcal{E} = \mathcal{E}_x\hat{x} + \mathcal{E}_y\hat{y} + \mathcal{E}_z\hat{z}$. According to Eqn. (7), $\mathcal{E}_x$ only induces a current along $\hat{x}$ (otherwise, the integral is over an odd function and vanishes): $j_x = \sigma_{xx}\mathcal{E}_x$. Likewise, $\mathcal{E}_y$ induces a current along $\hat{y}$; and $\mathcal{E}_z$ induces a current along $\hat{z}$. This corresponds to situation (a) listed above.

If $\sigma_{xx} = \sigma_{yy}$ and the measurement is performed within the xy plane, the induced current must be along the electric field, regardless of the direction along which the electric field is applied, and there is no Hall response. This corresponds to situation (b).

Finally, if the electric field $\mathcal{E}$ is applied off-axis to the main crystallographic directions and the measurement is not performed within the tetragonal plane, a Hall-like linear-in-$\mathcal{E}$ signal might indeed appear. This corresponds to situation (c). However, its temperature dependence should track the anisotropy of the resistivity of the probed directions.

In Supplementary Fig. S6, we plot the temperature dependence of $\rho_c/\rho_{c'}$ together with the one of the spontaneous Hall resistivity. The curves are very different. In particular, the relative change of the resistivity anisotropy is entirely negligible compared to that of the (Berry curvature-related) spontaneous Hall signal, making it extremely unlikely that what we observe here is a trivial anisotropy effect.

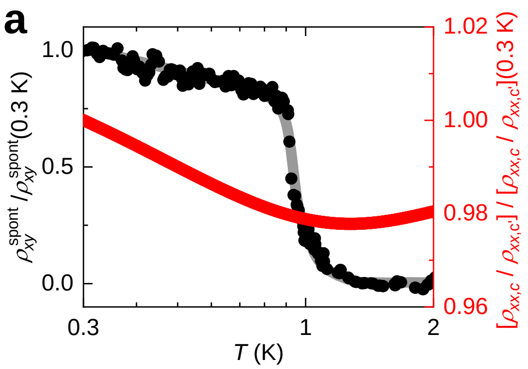

Fig. S6. **Hall-like contribution from anisotropy.** **a**, Comparison of the temperature dependence of the spontaneous Hall effect (black, left axis) and the anisotropy of the longitudinal resistivities $\rho_c(T)/\rho_{c'}(T)$ (red, right axis), both normalized at 0.3 K for better comparability. **b**, Curves of panel **a** are plotted on the same scale. This shows that the anisotropy contribution to the Hall-like signal is completely negligible.

At higher temperatures, however, the anisotropy acquires some temperature dependence, which can influence the measured Hall resistance. Indeed, for data taken between 2 K and 300 K in a ^{4}He cryostat, the scaling assumed for the contact misalignment correction in our dilution refrigerator measurements (Supplementary Discussion 2) no longer works perfectly (Supplementary Fig. S7a). A more elaborate analysis, taking into account that the measured Hall signal can contain contact misalignment contributions from both R_{xx} and R_{yy}, gives better overlap (Supplementary Fig. S7b). As below 2 K the temperature dependence of the anisotropy is negligible on the scale of the spontaneous Hall signal (Supplementary Fig. S6), the simplified correction with only αR_{xx} is sufficient for the data presented in the main text.

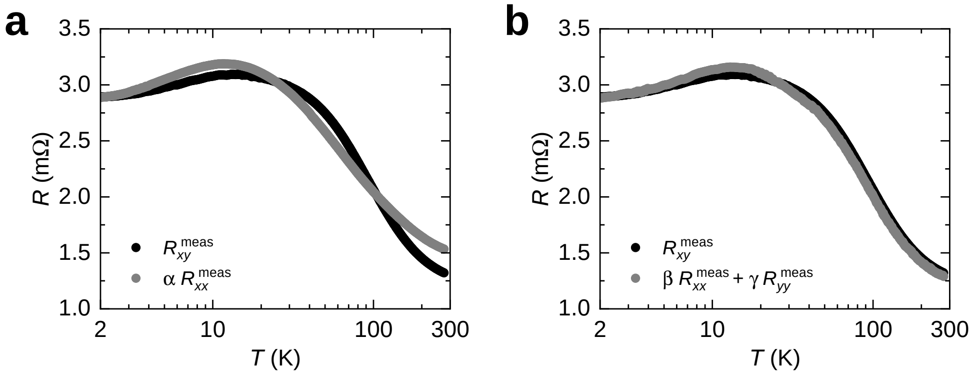

Fig. S7. **Contact misalignment correction at temperatures above 2 K. a**, Temperature-dependent resistance at ambient pressure, measured in a ^{4}He cryostat above 2 K, on the Hall contacts (black) and the resistivity contacts (gray), with the latter scaled by the factor $\alpha = 0.134$ determined in the dilution refrigerator measurements by minimizing the difference between the two curves just above the onset of the spontaneous Hall signal. **b**, More elaborate analysis, taking the electrical resistivity anisotropy of $CeRu_4Sn_6$ into account in the Hall contact misalignment correction: The coefficients $\beta = -0.381$ and $\gamma = 0.626$ of the linear superposition $\beta R_{xx} + \gamma R_{yy}$ were obtained such that the mean deviation from R_{xy}^{meas} is minimized.

Influence of resistivity anisotropy on spontaneous Hall conductivity. In materials with anisotropic resistivity, notably $\rho_{xx} \neq \rho_{yy}$, inversion of the resistivity matrix yields

$$\sigma_{xx} = \frac{\rho_{yy}}{\rho_{xx}\rho_{yy} + \rho_{xy}^2} \quad \text{and} \quad \sigma_{xy} = \frac{\rho_{xy}}{\rho_{xx}\rho_{yy} + \rho_{xy}^2} \tag{8}$$

for the longitudinal and Hall conductivities, respectively. For $\rho_{xx} = \rho_{yy}$, this simplifies to

$$\sigma_{xx} = \frac{\rho_{xx}}{\rho_{xx}^2 + \rho_{xy}^2} \quad \text{and} \quad \sigma_{xy} = \frac{\rho_{xy}}{\rho_{xx}^2 + \rho_{xy}^2} \ , \tag{9}$$

the relations used to analyze the data in the main text.

To determine the x-y anisotropy of the sample used for the pressure-dependent spontaneous Hall effect measurements in the main text (a bar-shaped sample along x), we added two contacts along the y direction (Hall measurements require only 6 contacts, resistivity anisotropy measurements 8). This guarantees that essentially the same sample volume is probed for both directions, at the price that the geometrical form factor for the y direction is somewhat ill-defined (the width is larger than the length). A rough estimate of the form factor results in an overall anisotropy $\rho_{yy}/\rho_{xx} \approx 1.4$ (Fig. S8a) and, with Eqn. 8, a somewhat

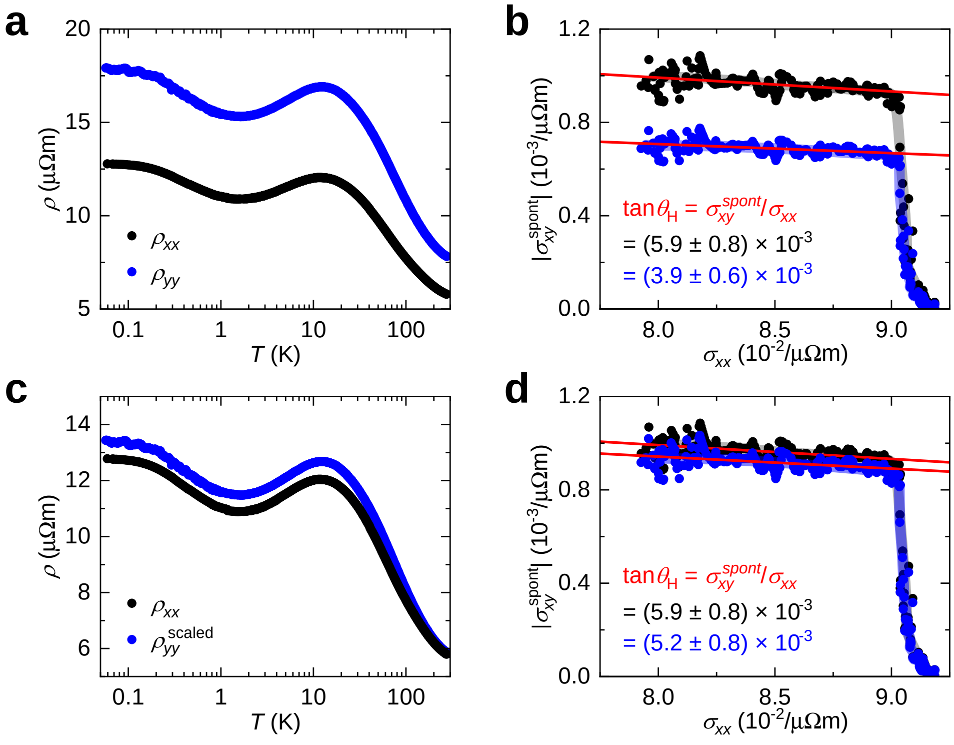

Fig. S8. **Influence of ρ_{xx} vs ρ_{yy} anisotropy on $\sigma_{xy}^{\text{spont}}$.** **a**, Temperature-dependent electrical resistivity along the x (black) and y (blue) directions of the same sample. The geometrical form factor of the y direction is somewhat ill-defined. **b**, Spontaneous Hall conductivity $\sigma_{xy}^{\text{spont}}$ vs longitudinal conductivity σ_{xx}, once calculated via Eqn. 9 as in the main text (black) and once via Eqn. 8 (blue), which takes the anisotropy of the longitudinal resitivities into account. **c**, **d** are the corresponding plots with ρ_{yy} scaled to ρ_{xx} a room temperature.

smaller Hall angle than found with Eqn. 9 (Fig. S8b). The shape of the spontaneous Hall signal is very similar in both cases. We also show the corresponding panels with ρ_{yy} scaled to ρ_{xx} at room temperature, which would still correspond to a plausible geometric form factor for ρ_{yy} (Fig. S8c,d). This reveals that the anisotropy has very little temperature dependence, in particular at low temperatures (as further discussed in Sect. "Hall contribution from resistivity anisotropy"), and that the spontaneous Hall data evaluated with both equations are now essentially the same. We conclude that the resistivity anisotropy of the investigated sample affects the spontaneous Hall response at best quantitatively, but will not change any of our conclusions.

We note that in [5], a somewhat larger anisotropy was found between ρ_c and $\rho_{c'}$, measured on two separate, bar-shaped and well-oriented samples. In the sample selected for the

pressure measurement, the alignment ($\rho_{xx} \approx \rho_c$ and $\rho_{yy} \approx \rho_{c'}$) was less precise, as were the form factors.

Magnetoresistance. For completeness, we also present low-temperature magnetoresistance data (Supplementary Fig. S9). In the field range of interest to the present work (below 100 mT), the magnetoresistance is extremely small (less than 0.25% at 100 mK and 100 mT) and featureless. This is corroborated by the featureless "normal" (i.e. antisymmetrized) Hall resistivity (Fig. 3e of original manuscript): it is simply linear in field in this field range, as expected for a small residual charge carrier concentration in a semimetal. At higher temperatures, It can thus be firmly ruled out that the spontaneous Hall features presented in our work are due to anomalies in the (longitudinal) magnetoresistivity.

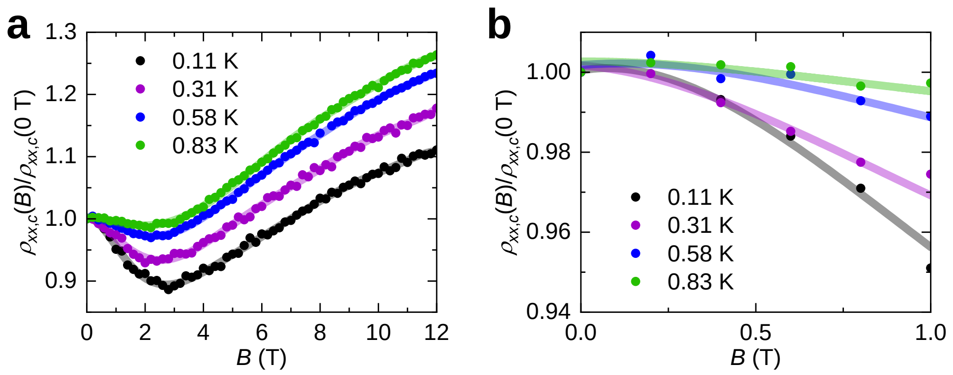

Fig. S9. **Magnetoresistance below 1 K.** **a**, Transverse magnetoresistance measured below 1 K on the transport sample from Figs. 2-4, with current along c and magnetic field along c'. **b**, Zoom into the low-field regime of panel a. Solid lines are guides to the eyes.

Hall mobility. The combined effect of the electrical resistivity and (normal) Hall coefficient is quantified by the Hall mobility, which, in a simple 1-band picture, is defined as $\mu_{\mathrm{H}} = R_{\mathrm{H}}/\rho$. It evolves smoothly with temperature and does not track the temperature dependence of the spontaneous Hall resistivity (Supplementary Fig. S10). This rules out sudden trivial (non-topological) current redistribution effects as the origin of the spontaneous Hall signal.

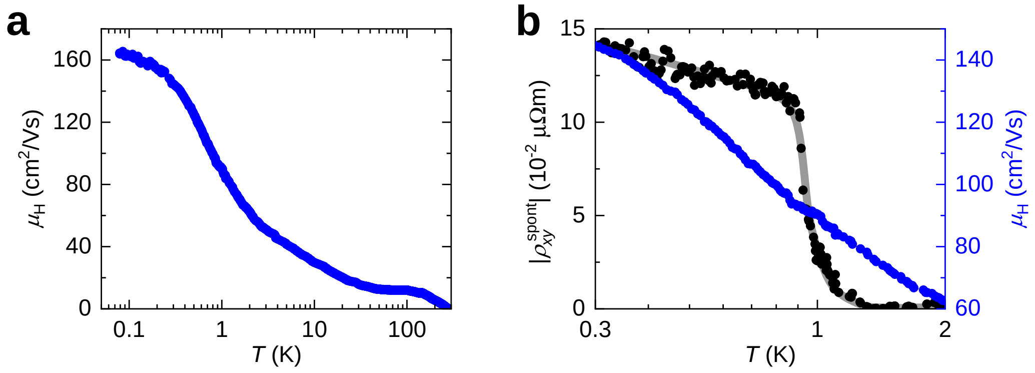

Fig. S10. **Hall mobility.** **a**, The Hall mobility $\mu_H = R_H/\rho$ in a simple 1-band picture varies smoothly with temperature. **b**, In particular, it does not track the temperature dependence of the spontaneous Hall resistivity ρ_{xy}^{spont}.

Supplementary Discussion 3: Measurement and analysis of specific heat data

Characterization of the AC calorimetry setup. As described in the Methods, the pressure-dependent specific heat measurements were performed in a pressure cell by AC calorimetry. Here, we provide a detailed characterization of the setup, and compare the data with our ambient pressure measurements with the relaxation time technique (Methods).

In a pressure cell, the time constant for thermal relaxation to the bath cannot be controlled, and tests are needed to demonstrate the functionality of the setup. For this purpose, we performed frequency response tests for each pressure at different temperatures before choosing our excitation frequencies, which ranged between $f = 5\,\text{Hz}$ at the lowest temperature and $f = 180\,\text{Hz}$ above 3 K. For the input load resistance of our setup ($\sim 5\,\Omega$), the gain of the used SR554 transformer preamplifier is essentially frequency-independent in the relevant frequency range (note that it is used to amplify the thermocouple voltage, which oscillates at twice the excitation frequency). Thus, no correction of the data for frequency-dependent gain deviations was needed. Exemplary frequency response curves are shown in Supplementary Fig. S11. The frequency response depends on the characteristic time constants τ_1 (thermal relaxation to the bath) and τ_2 (internal time scale of the sample assembly) of the system and can be described by [6]

$$F(\omega) = \left[1 + \frac{1}{\omega^2\tau_1^2} + \omega^2\tau_2^2\right]^{-1/2} . \qquad (10)$$

For an ideal AC calorimetry measurement, the frequency should be chosen such that $(\omega\tau_1)^2 >> 1$ and $(\omega\tau_2)^2 << 1$. The frequency that meets these criteria best (also called optimal frequency ω_{opt}) corresponds to the frequency at which $F(\omega)$ is maximal (ideally $F(\omega) \approx 1$). All our frequency response curves show a broad maximum, suggesting the above criteria are fulfilled. This was further confirmed by fitting our frequency response data to Eqn. 10, which allows us to extract the time constants for our system. The fits (solid lines in Supplementary Fig. S11) describe the experimental data very well and confirm that $\tau_2 << \tau_1$ is fulfilled in the entire temperature and pressure range relevant for our experiments. For example, at $p = 18.2\,\mathrm{kbar}$ the fits yield $\tau_1 = (0.146 \pm 0.015)\,\mathrm{s}$ and $\tau_2 = (0.0052 \pm 0.0008)\,\mathrm{s}$ at $0.1\,\mathrm{K}$ and $\tau_1 = (0.028 \pm 0.0009)\,\mathrm{s}$ and $\tau_2 = (0.0007 \pm 0.0001)\,\mathrm{s}$ at $1\,\mathrm{K}$. With the respective excitation frequencies this results in $(\omega\tau_1)^2 \approx 22 >> 1$ and $(\omega\tau_2)^2 \approx 0.03 << 1$ at $T = 0.1\,\mathrm{K}$ and $(\omega\tau_1)^2 \approx 118 >> 1$ and in $(\omega\tau_2)^2 \approx 0.08 << 1$ at $T = 1\,\mathrm{K}$. Note that we take the ω appearing in Eqn. 10 to be the excitation frequency, whereas in [6], it is the second harmonic of the excitation frequency. This difference has no consequence, except that, for a quantitative comparison with [6], our time constants should be divided by 2.

The knowledge of τ_1 and τ_2 can be used to calculate an error in the determination of absolute specific heat values, namely when $F(\omega_{\mathrm{opt}})$ deviates from the ideal value of 1. In our case, this error was typically less than 6% in the relevant temperature range (as seen from the maxima of the solid lines in Supplementary Fig. S11). Furthermore, we corrected our AC specific heat data as a function of temperature for this small error by incorporating the measured phase shift ϕ of the temperature oscillations into the analysis. Ideally, this phase shift is close to $\pi/2$. Small deviations from this value can be accounted for by a factor $\sin\phi$, which is approximately equal to the correction factor $F(\omega_{\mathrm{exc}})$ at a fixed frequency.

In addition, the excitation frequency should be sufficiently low for the thermocouple to detect the temperature oscillations created by the AC excitation. For this purpose, the distance between heater and thermocouple across the sample should be smaller than the thermal diffusion length. Our estimate for the ambient pressure thermal diffusion length at $0.4\,\mathrm{K}$, using our specific heat data measured in the PPMS and published thermal conductivity data [7], $0.363\,\mathrm{J/(mol\,K)}$ and $0.033\,\mathrm{J/(K\,m)}$ at $0.4\,\mathrm{K}$, we estimate a thermal diffusivity of $1.26 \cdot 10^{-5}\,\mathrm{m/s^2}$. For an excitation frequency of $f = 5\,\mathrm{Hz}$ and thus temperature oscillations at $2f = 10\,\mathrm{Hz}$, the thermal diffusion length $l_0 = \sqrt{(D/2f\pi)}$ is $0.63\,\mathrm{mm}$, which is indeed longer than the distance (about $0.28\,\mathrm{mm}$) between the thermocouple and the closest heater

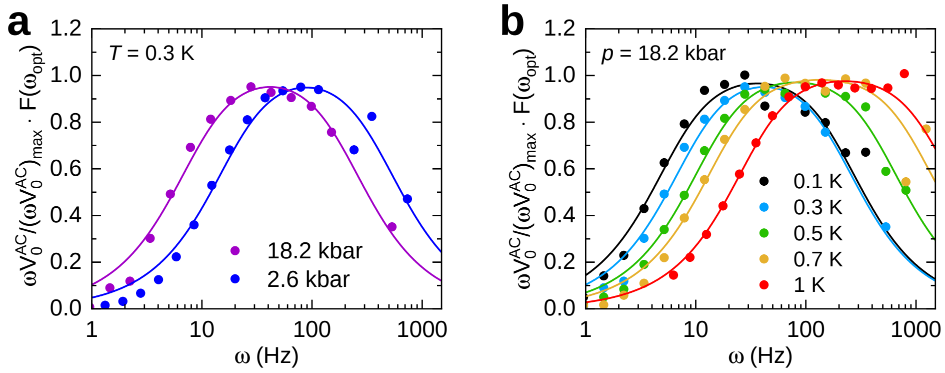

Fig. S11. **Frequency response of the thermocouple voltage.** **a**, Product of the amplitude of the thermocouple's oscillating voltage V_0^{AC} and (angular) frequency $\omega = 2\pi f$, normalized to its maximum value, and scaled by the maximum of the calculated frequency response $F(\omega_{\mathrm{opt}})$, plotted as function of ω at $T = 0.3\,\mathrm{K}$, for 2.6 kbar and 18.2 kbar (the smallest and largest pressure of our AC C_p experiments). **b**, Same quantity as in **a**, for different temperatures at 18.2 kbar. Solid lines correspond to fits using Eqn. 10.

contact in our sample assembly. The entire sample had the following dimensions: length × width × thickness = $(2 \times 0.65 \times 0.56)\,\mathrm{mm}^3$. Because the sample length is larger than l_0 at 10 Hz, it may seem preferable to use even significantly lower excitation frequencies. However, this would not only have taken us too far away from the optimal excitation frequency determined from the frequency response tests but may also have caused a gain reduction of our SR554 preamplifier (the lowest excitation frequency we used at the lowest temperatures was 5 Hz, where the gain reduction is still negligible). As a consequence, the AC calorimetry measurement may not have probed the entire sample volume. This will affect the absolute value of the specific heat (which is difficult to extract anyway and was not used in our work) but should not have a major impact on the observed pressure and temperature dependencies.

In Fig. 3b, we plot the AC data at various pressures together with an ambient pressure curve obtained with the relaxation time technique. Because the AC technique does not produce specific heat data in absolute units, these data were scaled at low temperatures to the ambient pressure data. The satisfactory overall agreement supports the good functionality of our AC specific heat measurement in the pressure cell. Of course, this is a challenging technique that is often merely used to detect sharp features such as phase transitions. Here, we are pushing the technique a step further, to resolve trends in the data as a function of

pressure.

As seen in Fig. 3b, the agreement between the lowest pressure AC curve and our ambient pressure relaxation time curve is very good at low temperatures. Here, the AC technique appears to be particularly reliable. At higher temperatures, larger deviations are observed. A likely source of systematic errors is that we used generic thermopower curves from the literature to convert the measured voltage of our Chromel/Au-0.07at%Fe thermocouple to temperature (Methods), one below and one above 1 K. Apparently, this works much better at low temperatures than at high temperatures. In addition, the sensitivity of the thermocouple decreases with increasing temperatures ($S(T)$ dependence flattens at higher T), which will further reduce the precision. However, since these thermocouples have rather weak pressure dependence [8, 9], we expect the trends under pressure to be more robust than the temperature dependence as such.

Finally, we show the pressure dependencies of the time constants and of the correction factor $F(\omega_{\rm exc})$ at $T = 0.3\,$K, where the specific heat shows a broad maximum and exhibits the largest pressure dependence (Supplementary Fig. S12). Whereas τ_1 and τ_2 show a modest increase with pressure, $F(\omega_{\rm exc})$ is essentially pressure-independent, indicating that the pressure dependencies we measure are intrinsic. A simple estimate illustrates this further.

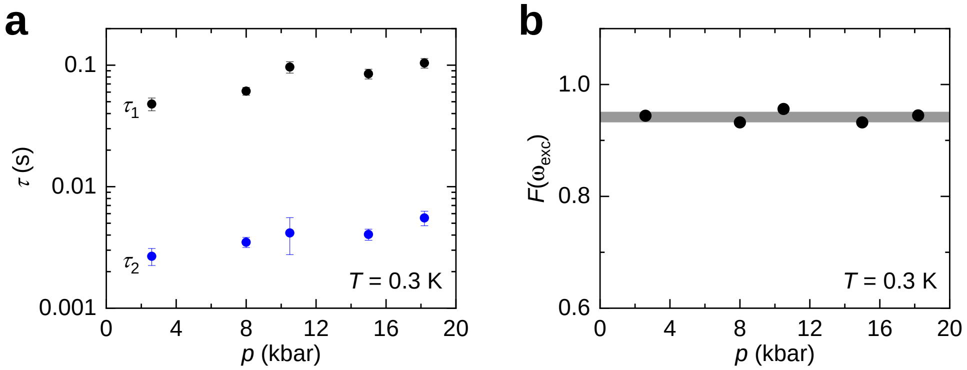

Fig. S12. **Pressure dependencies of the time constants and of the correction factor at 0.3 K.** **a**, Characteristic time constants τ_1 (thermal relaxation to the bath) and τ_2 (internal time scale of the sample assembly) as function of pressure. The error bars represent the standard error of the fits to Eqn. 10. **b**, Pressure dependence of the correction factor $F(\omega_{\rm exc})$, calculated from Eqn. 10 using the time constants in **a** and the respective excitation frequency. The solid line is a guide to the eyes, emphasizing that $F(\omega_{\rm exc})$ is essentially pressure independent.

Under proper measurement conditions, the specific heat C is inversely proportional to the measured temperature oscillation amplitude $T_0^{\mathrm{AC}} \approx P/(2\omega C) \cdot F(\omega)$, where P is the applied heating power and ω the chosen excitation frequency. Let us now assume that, at a given temperature, the entire observed pressure dependence of this amplitude is extrinsic, i.e., due to a pressure dependence of $F(\omega)$. At 0.3 K, where C varies by almost 40% under pressure, a similar variation of $F(\omega)$ should be seen (which is clearly not the case, Supplementary Fig. S12b); this would require both time constants to be approximately equal, in stark contrast with our observation (Supplementary Fig. S12a).

Phonon contribution to the specific heat. Acoustic phonons and Weyl fermions both contribute a cubic-in-temperature term to the specific heat. Thus, in general, an unambiguous separation is difficult. However, the situation in heavy fermion compounds is fortuitous. The electronic specific heat contribution gets strongly enhanced only at low temperatures as Kondo (lattice) coherence is established (typically well below the single-ion Kondo scale T_{K}). At higher temperatures, phonons generally dominate and can thus be directly measured. Furthermore, a noninteracting "reference" material is available for most heavy fermion compounds. In the case of Ce-based compounds, this is the associated La-based compound, which differs from the heavy fermion compound only by the absence of one $4f$ electron (and, of course, one less proton in the nucleus). While this removes the Kondo physics, it is expected to leave the phonon system largely unaffected.

In our case, this reference material is $LaRu_4Sn_6$, which indeed shows the expected C/T vs T^2 behavior down to low temperatures [10]. That a Debye contribution can also be directly revealed in $CeRu_4Sn_6$ is shown exemplarily (for the 10.5 kbar data) in Supplementary Fig. S14b, where the normal (Debye-like) specific heat holds down to about 8 K. This contribution is slightly pressure-dependent. Thus, for each isobar, a fit analogous to that in Supplementary Fig. S14b was made and subtracted from the data to obtain the electronic contribution C_{el}/T plotted in Fig. 3b. Most importantly, however, this subtraction leaves the low-temperature data of interest to us here almost unaffected. This can be seen by comparing the total specific heat coefficient data (Supplementary Fig. S13) with the electronic specific heat coefficient data of Fig. 3b. Both data sets are essentially the same; only above about 4 K, a small (phonon-dominated) increase of C/T is seen. Thus, despite the same functional form of the specific heat of acoustic phonons and Weyl fermions, the phonon subtraction is well-controlled and unlikely to induce sizable errors.

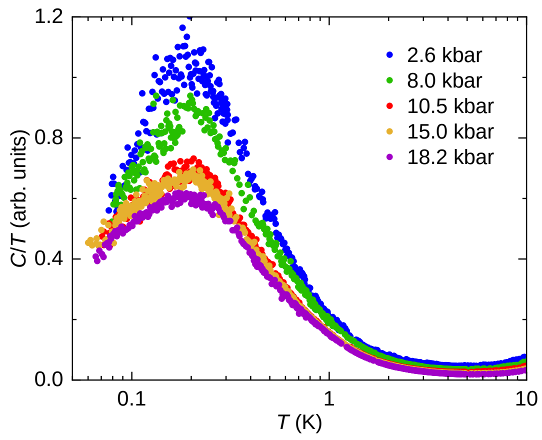

Fig. S13. **Influence of phonons on the low-temperature specific heat.** Temperature dependence of the total specific heat coefficient C/T at various fixed pressures. The curves can hardly be distinguished from the electronic specific heat coefficient $C_{\rm el}/T$ plotted in Fig. 3b (where the phonon contribution was subtracted). Only above about 4 K, a small (phonon-dominated) increase of C/T is seen.

Disentangling non-Fermi liquid and Weyl contributions. At each pressure, the electronic specific heat $C_{\rm el}(T)$ of $CeRu_4Sn_6$ is determined by subtracting a Debye-like phonon contribution $C_{\rm ph}(T) = \beta T^3$ from the total measured specific heat $C(T)$, as shown exemplarily for the 10.5 kbar data in Supplementary Fig. S14a-c. $C_{\rm el}(T)$ can be modeled as a crossover between the specific heat of a Weyl-Kondo semimetal [11, 12]

$$C_{\rm Weyl}(T) = \Gamma T^3 = \frac{7\pi^2 V}{30} k_{\rm B} \left(\frac{k_{\rm B} T}{\hbar v_{\rm Weyl}} \right)^3 \tag{11}$$

at low temperatures and that of a quantum critical system

$$C_{\rm qc}(T) = BT^{-b} \tag{12}$$

at high temperatures, where $v_{\rm Weyl}$ is the Weyl velocity, V is the sample volume, and B and b are constants. To show this explicitly, we use the crossover function

$$f_{\rm cross}(x) = f_2(x) - \frac{f_2(x) - f_1(x)}{1 + (x/x_0)^a} \tag{13}$$

introduced in [13] to phenomenologically describe the change of Hall coefficient isotherms across a QCP at the critical tuning parameter value x_0, where a is the sharpness of the crossover between $f_1(x) = R_{H,1}$ and $f_2(x) = R_{H,2}$. The function $f_{\rm cross}$ smoothly interpolates

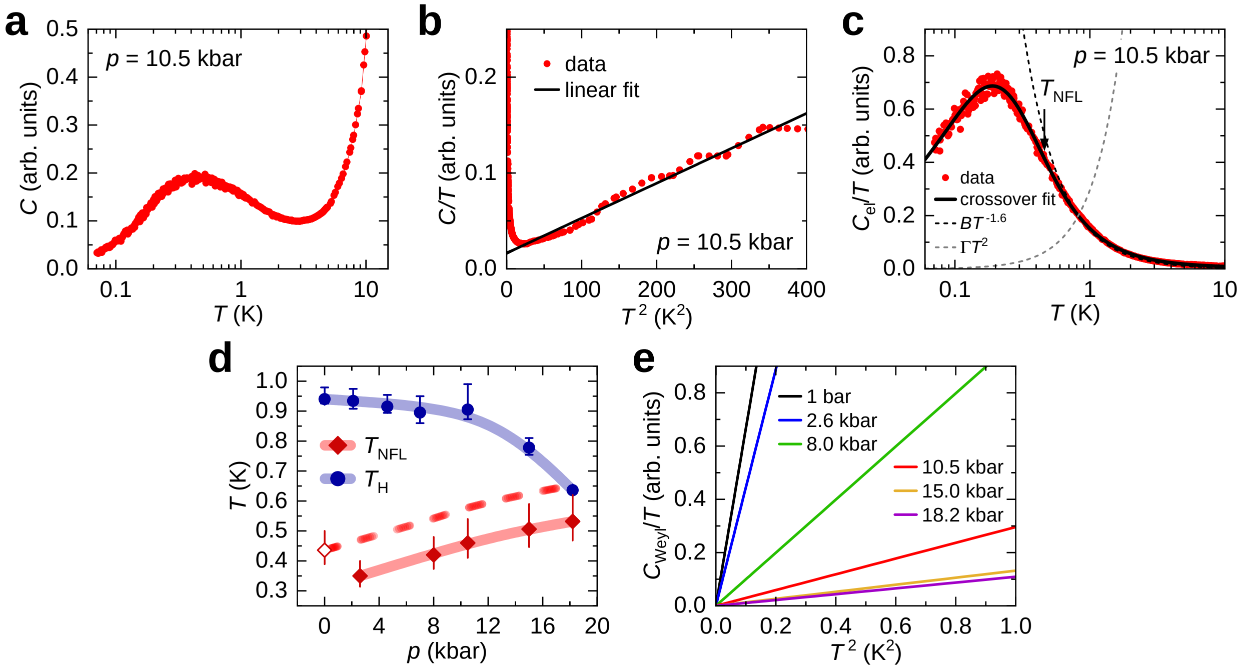

Fig. S14. **Analysis of temperature-dependent specific heat isobars.** **a**, Temperature dependence of the specific heat $C(T)$ of $CeRu_4Sn_6$ at 10.5 kbar. **b**, Specific heat coefficient C/T at 10.5 kbar plotted vs T^2, together with a Debye fit made between 8.5 K and 20 K to estimate the phonon background. **c**, Temperature-dependent electronic specific heat coefficient C_{el}/T at 10.5 kbar, with phenomenological fit (black solid line) describing a crossover between a Weyl-Kondo contribution (gray dashed line) at low temperatures and a quantum critical contribution (black dashed line) at high temperatures (see text). **d**, Temperature-pressure phase diagram of $CeRu_4Sn_6$ in zero magnetic field, defined by the onset temperature T_{H} of the spontaneous Hall effect (blue dots), where $\rho_{xy}^{\mathrm{spont}}$ reaches 50% (±15% within the error bars) of the crossover height (see Supplementary Fig. S2c), and the lower bound of the quantum critical fan T_{NFL} (red diamonds) defined by $f_{\mathrm{Hill}} = 0.2$ (±0.05 within the vertical bars, see text) as extracted from the crossover fits as in panel c. Solid and dashed lines are guides to the eyes. The results are consistent with the cartoon of an emergent phase shown in Fig. 4b. **e**, Weyl-Kondo contribution obtained from the crossover fits, plotted as C_{Weyl}/T as function of T^2.

between the functions f_1 and f_2, i.e., $f_{\mathrm{cross}} = f_1$ for $x \ll x_0$ and $f_{\mathrm{cross}} = f_2$ for $x \gg x_0$. For the special case $R_{H,1} = 1$ and $R_{H,2} = 0$, this function takes the form

$$f_{\mathrm{Hill}}(x) = \frac{1}{1 + (x/x_0)^a}\ , \tag{14}$$

which is 1 at $x = 0$, zero at $x \to \infty$, and 1/2 at $x = x_0$.

Here, we use $f_{\rm cross}(x)$ to describe thermal crossovers in our specific heat isobars, i.e., $x = T$, $x_0 = T_0$, $f_1(x) = C_{\rm Weyl}(T)$, and $f_2(x) = C_{\rm qc}(T)$. The quality of the fits is good for all pressures (Fig. 3b). The Weyl contributions $C_{\rm Weyl}(T)$ extracted from fits to the different isobars are shown in Supplementary Fig. S14, the pressure-dependent Weyl velocity $v_{\rm Weyl}$ in Fig. 3c. We further extract $T_{\rm NFL}$ as a measure of the "lower bound" of the quantum critical fan, using the following criterion: at $T_{\rm NFL}$, $f_{\rm Hill} = 0.2$, which means that $C_{\rm el}(T)$ deviates by roughly 20% from the power law contribution $C_{\rm qc}(T)$. The pressure dependence of $T_{\rm NFL}$ is shown in Supplementary Fig. S14d. The vertical bars represent the criteria $f_{\rm Hill} = 0.15$ and 0.25. We find the following trends: (i) The temperature $T_{\rm H}$, a measure of the *upper* bound of the Weyl-Kondo semimetal (with $C_{\rm Weyl}(T) = \Gamma T^3$ behavior), *de*creases as we move away from quantum criticality with increasing pressure, and (ii) $T_{\rm NFL}$, a measure of the *lower* bound of the quantum critical fan (with $C_{\rm qc}(T) = BT^{-b}$ behavior), *in*creases with increasing pressure. The results of the fits, together with $T_{\rm H}$ and $T_{\rm NFL}$, are summarized in Table II.

TABLE II. Results from crossover fits of temperature-dependent specific heat curves at various pressures. b was fixed to -1.6. As the absolute values of specific heat data taken by AC calorimetry in a pressure cell have only limited accuracy, we provide the results only in arb. units. For clarity, we nevertheless write out the other units.

p [kbar]	Γ [arb. units/K^3]	B [arb. units/K$^{-0.4}$]	T_0 [K]	a	$T_{\rm NFL}$ [K]	$T_{\rm H}$ [K]
0.001	6.66 ± 0.91	0.201 ± 0.010	0.252 ± 0.006	2.53 ± 0.02	0.44	0.94
2.6	4.45 ± 1.01	0.157 ± 0.007	0.211 ± 0.004	2.77 ± 0.03	0.35	0.93
8.0	1.00 ± 0.19	0.166 ± 0.004	0.241 ± 0.003	2.50 ± 0.02	0.42	0.90
10.5	0.30 ± 0.07	0.145 ± 0.002	0.258 ± 0.003	2.36 ± 0.01	0.46	0.91
15.0	0.13 ± 0.04	0.151 ± 0.002	0.273 ± 0.002	2.25 ± 0.01	0.51	0.78
18.2	0.11 ± 0.02	0.144 ± 0.001	0.285 ± 0.002	2.21 ± 0.01	0.53	0.64

To test whether the parameters extracted from the AC specific heat data are robust against the systematic errors of the AC calorimetry setup (see Sect. "Characterization of the AC calorimetry setup" above), we also fit the ambient pressure data (obtained with the "standard" relaxation time technique) with the same crossover function. For a good fit

quality, we have to limit the fitting range to below 2 K, which confirms that the temperature dependence of the AC data is somewhat distorted due to systematic errors at high temperatures. The resulting Weyl velocity v_{Weyl} is in good agreement with the pressure cell data (Fig. 3c), which is expected as Weyl physics contributes mostly at low temperatures. The value extracted for T_{NFL} is somewhat larger (Fig. S14d), in agreement with the larger uncertainties at high temperatures. As we trust the pressure dependence of T_{NFL} (red shaded curve in Fig. S14) more than the absolute value, we propose that a similar curve anchored to the new zero-pressure data point (red dashed curve) describes the situation more accurately. This comparison supports the robustness of our analysis in revealing the pressure trends of both v_{Weyl} and T_{NFL}.

Absence of a thermal phase transition. It is important to note that, in the parameter window accessed here, no thermal phase transition is experimentally detected. A thermal phase transition gives a clear signature in specific heat (a "lambda-type" anomaly for a continuous one) and, as this is not observed in our data, we can firmly rule it out. Instead, our quantitative analysis of the heat capacity data (Sect. "Disentangling non-Fermi liquid and Weyl contributions" above) captures the expected pressure effect on an emergent Weyl-Kondo semimetal nucleating out of quantum critical fluctuations, namely the concomitant suppression of the signatures of quantum criticality and of the emergent Weyl-Kondo semimetal. Also on theoretical grounds, no thermal phase transition is expected because: (i) the emergent Weyl-Kondo semimetal is a topological phase related to the electronic structure and as such characterized by topological indices and not an order parameter; (ii) at zero pressure and zero magnetic field, the system is ("genuinely") quantum critical; (iii) as neither pressure nor magnetic field (as shown previously [14]) induces an ordered phase, we hypothesize that order (likely of antiferromagnetic nature) appears under "negative" pressure, a hypothesis that could, for instance, be tested by partially substituting Sn by Pb.

Nuclear Schottky contribution to the specific heat. The low-temperature feature in the specific heat analyzed above cannot be attributed to a nuclear Schottky contribution. The nuclear Schottky anomaly evoked in [15] is situated at much lower temperatures (outside the accessed temperature range) such that only its high-temperature tail is observed (see Supplementary Fig. S15). This is corroborated by tabulated resonance frequencies of the hyperfine structures of Sn [16] and Ru [17] (all stable Ce isotopes have no nuclear moment). In zero magnetic field, these correspond to energy splittings in the range of μeV, which

will result in nuclear Schottky peaks centered at 50 mK or below. Finite fields enhance the splitting, but even up to the highest field of 10 T applied in [15] the maximum of the Schottky anomaly does not move into the accessed temperature range. That the C/T upturn at higher temperatures, which we attribute to quantum criticality, is less pronounced for the polycrystal studied in [15] than for our single crystal might be due to a small stoichiometry difference that shifts the polycrystal slightly away from the quantum critical point.

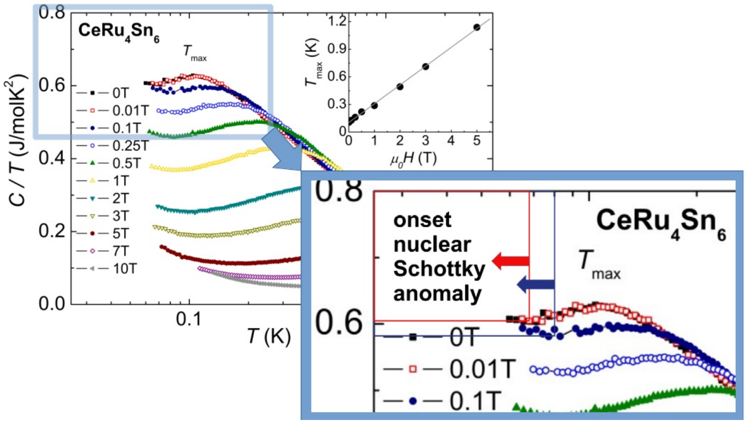

Fig. S15. **Nuclear Schottky anomaly.** We here replot Fig. 4 of Brüning et al. [15] (adapted with permission, copyrighted by the American Physical Society) and zoom into the low-temperature part to illustrate that the nuclear Schottky anomaly sets in only below about 65 mK at 0 T and 0.01 T, and below 80 mK at 0.1 T . It does thus not correspond to the "anomaly" of interest to us here.

Supplementary Discussion 4: Pressure effect on the electrical resistivity

At ambient pressure, the (longitudinal) electrical resistivity ρ_{xx} of $CeRu_4Sn_6$ is typical of a semimetal, with a modest increase with decreasing temperature (Fig. 1b). This assignment is further supported by the saturation of the (normal) Hall coefficient at the lowest temperatures (inset Fig. 1b). However, with increasing pressure, the increase of ρ_{xx} with decreasing temperature is strongly enhanced (Supplementary Fig. S16a), consistent with previously published results [18]. It is the strong increase of ρ_{xx} below 1 K, better seen on a linear y scale (Supplementary Fig. S16b), that makes the contact misalignment correction explained in Supplementary Discussion 2 difficult at large pressures. There is no evidence for a pressure-induced phase transition, in agreement with the specific heat data.

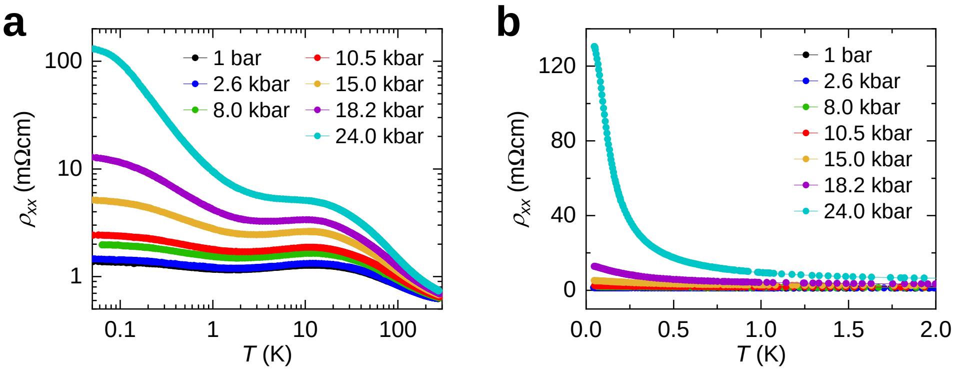

Fig. S16. **Electrical resistivity of $CeRu_4Sn_6$ under pressure.** **a**, Temperature-dependent (longitudinal) electrical resistivity isobars $\rho_{xx}(T)$ with current along c, used for the contact misalignment correction described in Supplementary Discussion 2, on a double-logarithmic scale. **b**, Detailed view of the low-temperature part of $\rho_{xx}(T)$ on a linear scale.

It is also interesting to ask what this pressure dependence implies for the ground state of $CeRu_4Sn_6$. In Kondo insulators, the width of the hybridization gap typically increases with pressure (at least up to modest pressures), as seen for instance in $Ce_3Bi_4Pt_3$ [19] and FeSi [20]. This suggests that the topologically trivial background in $CeRu_4Sn_6$, i.e. the bandstructure out of which the emergent topological semimetal evidenced here nucleates, features a narrow hybridization gap, which opens further with pressure. Indeed, the onset of hybridization gap formation in $CeRu_4Sn_6$ was evidenced in a combined optical conductivity and ab initio-based dynamical mean field theory study [21], even though the limited energy and temperature ranges accessed did not allow to conclude on the material's ground state. In Supplementary Discussion 7 we discuss the normal (non-emergent) Weyl-Kondo semimetal $Ce_3Bi_4Pd_3$, which also appears to exhibit a narrow hybridization gap as background to the topological semimetal state [22].

Supplementary Discussion 5: Details on the theoretical model and computations

In this section, we provide further details of our theoretical work on a Weyl-Kondo semimetal that develops out of the fluctuations associated with a quantum critical point (QCP). The QCP we study is caused by a dynamical competition between the Kondo coupling and the Ruderman-Kittel-Kasuya-Yosida (RKKY) interaction. As presented in the main text (Fig. 5), we establish a Kondo-destruction QCP and, utilizing the formalism of

symmetry constraints placed on Green's function eigenvectors [23], we identify a non-Fermi liquid type of Weyl-Kondo semimetal that it drives.

We focus on a topological heavy fermion model, which is specified by an Anderson lattice Hamiltonian as

$$\mathcal{H} = \mathcal{H}_c + \mathcal{H}_{cf} + \mathcal{H}_f \ . \tag{15}$$

The part $\mathcal{H}_c$ represents a noninteracting tight-binding model of the conduction electrons. It is defined on a stacked kagome lattice, and takes the form of $\mathcal{H}_c = \sum c^\dagger_{\boldsymbol{k},\sigma}[H_{0,\sigma}(k) - \mu\mathbb{I}]c_{\boldsymbol{k},\sigma}$. Here, $H_{0,\uparrow} = \zeta(k)$, $H_{0,\downarrow} = \zeta^*(-k)$ and $c_{\boldsymbol{k},\sigma} = (c_{A,\boldsymbol{k},\sigma}\ c_{B,\boldsymbol{k},\sigma}\ c_{C,\boldsymbol{k},\sigma})^T$, where the three sites of the kagome lattice's unit cell are marked by A, B, C. We can express $\zeta(k) = \sum_{a=1}^4 \zeta_a(k)$, where $\zeta_1(k) = -t_2 M_1$, $\zeta_2(k) = [t_1 - t_{z,2}\cos(k_z)]M_2$, $\zeta_3(k) = [i\gamma + i\gamma_z\cos(k_z)]M_3$, and $\zeta_4(k) = -t_z\cos(k_z)\mathbb{I}$, and the matrices are

$$M_1 = \begin{pmatrix} 0 & e^{i(-k_1-k_2)} + e^{ik_1} & 1 + e^{i(k_1-k_2)} \\ e^{i(k_1+k_2)} + e^{-ik_1} & 0 & e^{i(k_1+k_2)} + e^{-ik_2} \\ 1 + e^{-i(k_1-k_2)} & e^{-i(k_1+k_2)} + e^{ik_2} & 0 \end{pmatrix};$$

$$M_2 = \begin{pmatrix} 0 & (1+\alpha) + (1-\alpha)e^{-ik_2} & (1+\alpha)e^{ik_1} + (1-\alpha)e^{-ik_2} \\ (1+\alpha) + (1-\alpha)e^{ik_2} & 0 & (1+\alpha)e^{ik_1} + (1-\alpha) \\ (1+\alpha)e^{-ik_1} + (1-\alpha)e^{ik_2} & (1+\alpha)e^{-ik_1} + (1-\alpha) & 0 \end{pmatrix};$$

$$M_3 = \begin{pmatrix} 0 & 1 + e^{-ik_2} & -e^{ik_1} - e^{-ik_2} \\ -1 - e^{ik_2} & 0 & 1 + e^{ik_1} \\ e^{-ik_1} + e^{ik_2} & -1 - e^{-ik_1} & 0 \end{pmatrix}.$$

Here, $k_{1/2} = (k_x \pm k_y/\sqrt{3})/2$. Overall, $\mathcal{H}_c$ contains the nearest-neighbor and next-nearest neighbor hopping parameters within each kagome layer, t_1 and t_2; the nearest-neighbor and next-nearest-neighbor couplings across the kagome layers, t_z and $t_{z,2}$; and the intra-layer and inter-layer spin-orbit couplings, γ and γ_z. The M_y mirror and inversion symmetries are broken by the anisotropy parameter, α, which acts on the t and $t_{z,2}$ hopping terms. In our calculations, the representative parameters are $t_1 = 0.143, t_2 = 0.071, t_z = -0.086, t_{z,2} = 0.129, \gamma = 0.214, \gamma_z = 0.029, \alpha = 1.4$, and $\mu = -1$. The bandstructure of the conduction electrons contains Weyl nodes along the high-symmetry line $K - H$, at $\boldsymbol{k}^*$, and its symmetry-related counterparts, which are protected by a C_{3z} rotational symmetry [23]. The

Hamiltonian also contains a hybridization term, which is given by

$$\mathcal{H}_{cf} = V \sum_{i,\sigma} \left(c^{\dagger}_{i\sigma} f_{i\sigma} + h.c. \right) , \tag{16}$$

where $c^{\dagger}_{i\sigma}$ ($f^{\dagger}_{i\sigma}$) is the creation operator for the conduction c (local f) electron of spin σ at site i. Finally, the Hamiltonian for the f electrons is given by

$$\mathcal{H}_f = \sum_i \left(U n^f_{i,\uparrow} n^f_{i,\downarrow} - \mu_f \sum_\sigma n^f_{i,\sigma} \right) + \sum_{ij} I_{ij} \boldsymbol{S}_i \boldsymbol{S}_j . \tag{17}$$

It contains U and μ_f, which are the on-site repulsive Hubbard interaction and the energy level of the localized f electron, respectively, as well as an explicit antiferromagnetic (AF) RKKY coupling, I_{ij}. Here, $n^f_{i,\sigma} = f^{\dagger}_{i,\sigma} f_{i,\sigma}$ is the density operator for the localized f electrons of spin σ, and the corresponding spin operator is $\boldsymbol{S}_i = (f^{\dagger}_i \boldsymbol{\sigma} f_i)/2$. We adopt the parameters $V = 0.64$, $\mu_f = -0.2$, and $U = 0.4$. Note that the Weyl nodes appear at the Fermi energy as long as the Kondo energy scale T_{K} is small compared to the underlying conduction-electron bandwidth (see, e.g., [12, 24]).

The dynamical competition between the two kinds of interactions leads to a Kondo-destruction QCP (Fig. 5a), which involves a critical destruction of the quasiparticle excitations (Fig. 5b). We treat the dynamical competition using the extended dynamical mean field theory (EDMFT) [25]. This approach determines the correlation functions of the Anderson lattice model in terms of those of a self-consistent Bose-Fermi Anderson model

$$\begin{aligned} S_{\mathrm{BFA}} = & \int_0^\beta \left(\sum_\sigma f^{\dagger}_\sigma (\partial_\tau - \mu_f) f_\sigma + U(n^f_\uparrow + n^f_\downarrow) + h_{\mathrm{loc}} S^z \right) \\ & - \int_0^\beta d\tau d\tau' \left(\sum_\sigma f^{\dagger}_\sigma(\tau) V^2 \mathcal{G}_0(\tau - \tau') f_\sigma(\tau') \right) \\ & + \frac{1}{2} \sum_{a \in \{x,y,z\}} S^a(\tau) [\chi^a_0]^{-1}(\tau - \tau') S^a(\tau') , \end{aligned} \tag{18}$$

where $\beta = \frac{1}{T}$, $a \in \{x, y, z\}$ represents the spin directions, and h_{loc} is a static Weiss field. $\mathcal{G}_0$ and χ_0 denote the bare correlators of the fermionic and bosonic baths, respectively. They are determined by the self-consistent conditions

$$\begin{aligned} \left(\mathcal{G}_0^{-1}(i\omega_n) - \Sigma_c(i\omega_n) \right)^{-1} &= \sum_{\boldsymbol{k}} \frac{1}{i\omega_n - \epsilon_{\boldsymbol{k},c} - \Sigma_c(i\omega_n)} , \\ \left([\chi^a_0]^{-1}(i\Omega_n) + M^a(i\Omega) \right)^{-1} &= \sum_{\boldsymbol{q}} \frac{1}{I_{\boldsymbol{q}} + M^a(i\Omega_n)} , \end{aligned} \tag{19}$$

where $\epsilon_{\boldsymbol{k},c}$ are the eigenvalues of $\mathcal{H}_c$ and $I_{\boldsymbol{q}}$ is the Fourier transformation of I_{ij}. Here, $\Sigma_c(i\omega_n)$ and $M^a(i\Omega_n)$ are the self-energy and spin cumulant, respectively. In addition, a third self-consistency equation, $h_{\rm loc} = -\left(2I - [\chi_0^a]^{-1}(\Omega_n = 0)\right) m_{\rm AF}$, involves the static Weiss field $h_{\rm loc}$ and the ordered moment $m_{\rm AF}$.

As the strength of the RKKY interaction increases, the system undergoes an AF quantum phase transition. As shown in the main text, at the QCP, the spin correlations at the AF wavevector diverge. It obeys a dynamical Ω_n/T scaling, with a fractional (0.77) dynamical exponent (Fig. 5a).

We have performed an analytical continuation of $\Sigma_c(i\omega_n)$ to obtain the retarded self-energy. We used the N-point Padé approximants [26]

$$C_N(z) = A_N(z)/B_N(z)\ . \tag{20}$$

The polynomials A_N and B_N are of order $(N-1)/2$ and $(N-1)/2$ for N odd and $(N-2)/2$ and $N/2$ for N even. The first 60 Matsubara frequencies for $\beta = 800$ are used. The imaginary part of the resulting (retarded) conduction electron self-energy $-\Im\Sigma_c^R(\omega)$ follows a linear-in-ω dependence (Fig. 5b), which implies the vanishing of the quasiparticle residue. This signifies the suppression of the quasiparticle weight, which is associated with the destruction of the Kondo effect at the QCP.

As the wavevector is varied, the evolution of the Kondo-driven f-electron spectral function vs energy, for a very low temperature ($\beta = 800$), was presented in the main text (Fig. 5c,d). At a generic wavevector along the $K-H$ direction, there are two peaks in the spectral function vs energy. They intersect when the wavevector reaches $\boldsymbol{k}^*$ (Fig. 5c). In fact, at this wavevector, the two peaks completely overlap at $\boldsymbol{k}^*$ (Fig. 5d). This manifests the symmetry constraints enforced on Green's function eigenvectors, and defines a Weyl point.

The width of each spectral peak is linearly proportional to the peak energy measured with respect to the Fermi energy. In other words, the former is not small compared to the latter, and this specifies the non-Fermi liquid nature of the single-electron excitations. In this way, we have shown that the excitations near the Weyl node, which develops in the quantum critical regime, have a non-Fermi liquid form.

Note that, throughout this section, we have used dimensionless quantities and set $\hbar = k_{\rm B} = 1$.

Supplementary Discussion 6: Experiment vs theory

In the main text we have shown that our experimental and theoretical results together provide evidence for an emergent Weyl-Kondo semimetal phase in $CeRu_4Sn_6$. This overall understanding is our key result. Note that this phase is distinct from both the noninteracting Weyl semimetal and the "normal" (non-emergent) Weyl-Kondo semimetal.

The former can be seen as follows: In the Weyl-Kondo semimetal Hamiltonian of Eqn. 15, appropriate lattice symmetries will create Weyl nodes in the conduction electron part $\mathcal{H}_c$ (conveniently taken as noninteracting tight-binding model of the conduction electrons). These nodes, however, are generically far away from the Fermi energy and occur in broad noninteracting bands. Two conditions are needed for the Weyl nodes to appear at the Fermi energy: (i) The localized level (representing the $4f$ states of Ce) must be situated below the Fermi energy and the filling be commensurate, i.e., the same as for a Kondo insulator; and (ii) The Kondo interaction must be strong such that the noninteracting bands that host the topological crossing become part of the Kondo resonance, which is generically pinned to the Fermi energy. Point (i) represents a filling constraint in interacting settings. It also is consistent with the experimental situation. $CeRu_4Sn_6$ is, just as the first Weyl-Kondo semimetal $Ce_3Bi_4Pd_3$ discovered by some of us, a "failed" Kondo insulator, i.e., without the Weyl nodes, the density of states at the Fermi level would be gapped (in cubic Kondo insulators) or at least pseudogapped (in tetragonal Kondo insulators). For $Ce_3Bi_4Pd_3$ this was explicitly demonstrated in a high field study [22] where magnetic torque measurements revealed the characteristic feature of a magnetic field-induced Kondo insulator gap closing. Point (ii) also is consistent with the experimental situation, as evidenced by the extreme renormalization of the Weyl dispersion—we estimate the Weyl velocity to be at least 3 orders of magnitude smaller than the Dirac velocity of graphene. Such renormalizations are typical for Kondo systems (see, e.g., Fig. 2 of [27]), but would be rather unlikely in other settings. Even in the LDA + Gutzwiller scheme that partially captures the effect of interactions, the Weyl nodes detected in $CeRu_4Sn_6$ are still 15 meV (180 K) and 35 meV (420 K) away from the Fermi energy [28]. These are large energies compared to the width of the Kondo resonance (typically of the order of 10 K) and thus of the flat Weyl dispersion in a Weyl-Kondo semimetal. Thus, the signatures of interaction-driven topology (such as the large zero-field Hall response) are absent if $U = J = 0$.

The latter goes beyond the above argument, as the quantum critical state is distinct from both the $U = 0$ state and the "normal" (not quantum critical) finite U state. The emergent (quantum critical or "strange metal") Weyl-Kondo semimetal lacks well-defined Landau quasiparticles just as a topologically trivial strange metal state is expected to do (for recent experimental evidence, see e.g. [29]). A rather surprising theoretical result of our work is that, in this situation, Weyl nodes can nevertheless be defined, by examining the non-quasiparticle spectral functions and defining "generalized" non-Fermi liquid Weyl nodes as two collapsing spectral functions at a given position in momentum space (Fig. 5 of the main text).

Nevertheless, one may ask whether more detailed agreement between experiment and theory can reasonably be expected. $CeRu_4Sn_6$ is a strongly correlated electron compound, governed by the Kondo interaction. Despite considerable progress (for instance, for the related compound $Ce_3Bi_4Pd_3$, ab initio-based DFT & DMFT calculations have just been shown to be feasible down to a few tens of Kelvin by restricting the calculations to a subset of f orbitals [30]), a full ab initio treatment to temperatures below 1 K is beyond current possibilities. Therefore, predictions on the magnitude, angular dependence, or sign of the spontaneous Hall signal in $CeRu_4Sn_6$ are not available to date.

However, the value of the Weyl velocity v_{Weyl} was theoretically shown to be related to the slope of the cubic-in-T specific heat term via [12]

$$C_V = \frac{7\pi^2 V}{30} k_{\mathrm{B}} \left(\frac{k_{\mathrm{B}} T}{\hbar v_{\mathrm{Weyl}}} \right)^3 . \tag{21}$$

Within this model, the renormalization of the Weyl velocity is governed by the Kondo effect and thus by the magnitude of the Kondo temperature T_{K}. An established protocol to determine T_{K} is via the electronic entropy, which reaches $0.65 \ln 2$ per Ce atom at T_{K} [31]. Indeed, the Weyl velocity extracted from the slope of the T^3 dependence of the specific heat tracks the Kondo temperature extracted via the entropy (Supplementary Fig. S17). Furthest away from the quantum critical point (i.e. at the largest pressure), where the Weyl-Kondo semimetal is closest to "normal" (not quantum critical), the Weyl velocity and the Kondo temperature are largest, which is precisely what is expected from theory. This qualitative agreement strongly supports the theoretical interpretation of the experiments.

Furthermore, as shown previously [14] and discussed in detail in the main text, $CeRu_4Sn_6$ is genuinely quantum critical. The quantum criticality is amenable to theoretical descrip-

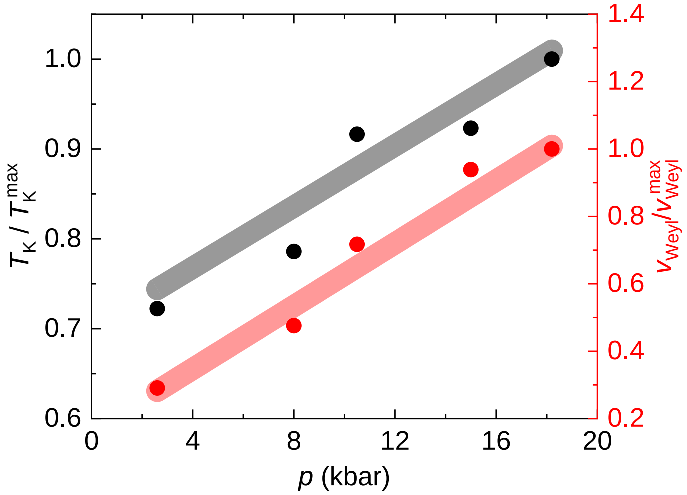

Fig. S17. **Kondo temperature and Weyl velocity vs pressure.** The smaller the Kondo temperature and the Weyl velocity, the stronger the renormalization, in agreement with the theoretical expectation within the Weyl-Kondo semimetal model [12]. Pressure thus tunes the material away from its quantum critical point, where the correlations are strongest. Solid lines are guides to the eyes.

tion, as described in the main text and Supplementary Discussion 5. Most notably, the dynamical ω/T scaling with a fractional exponent demonstrated here (Fig. 5a,b) is in excellent agreement with the scaling found for the dynamical structure factor of $CeRu_4Sn_6$ [14].

Supplementary Discussion 7: The Weyl-Kondo semimetal $Ce_3Bi_4Pd_3$

In the context of the present work, $Ce_3Bi_4Pd_3$ serves as a "normal" (not emergent) Weyl-Kondo semimetal reference material. It is an isostructural and isoelectronic sister compound of the well-known Kondo insulator $Ce_3Bi_4Pt_3$ [32, 33]. One way to evidence a Kondo insulator gap and estimate its magnitude is to suppress it with a magnetic field. Torque magnetometry, a sensitive probe for this gap suppression, revealed similar signatures for $Ce_3Bi_4Pt_3$ and $Ce_3Bi_4Pd_3$, albeit at distinctly different fields of about 40 T and 14 T, respectively [22]. Thus, $Ce_3Bi_4Pd_3$ could be seen as a narrower-gap analog of $Ce_3Bi_4Pt_3$, a view that was adopted in [34]. However, at low temperatures, topological states appear to form within the Kondo insulator gap of $Ce_3Bi_4Pd_3$. Key evidence for these states comes

from thermodynamic [11] and transport measurements [4]. That the topological states prevent a full gap from opening is also seen from (normal) Hall effect data [22, 34] (see also Supplementary Note 2 of [22]), which saturate to finite values in the low-temperature limit, as expected for a semimetal.

Supplementary Discussion 8: Longitudinal resistance change near onset of the spontaneous Hall effect

In dense, high-resolution data taken with low-temperature transformers, a small kink in R_{xx} (Supplementary Fig. S18a) is found to accompany the onset of the spontaneous Hall effect (Fig. 2a).

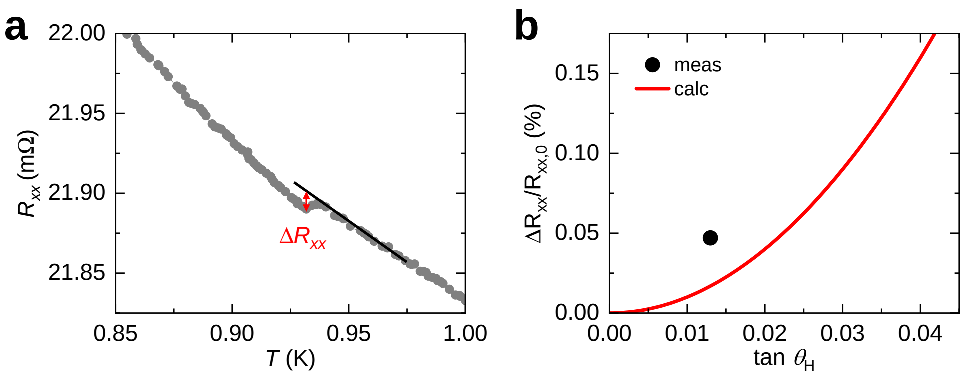

Fig. S18. **Longitudinal resistance change near the onset of the spontaneous Hall effect.** **a**, High-resolution measurement of the longitudinal electrical resistance R_{xx} of $CeRu_4Sn_6$ near the onset temperature of the spontaneous Hall effect. A tiny drop in R_{xx} is observed between 0.93 K and 0.94 K. To estimate the size of the change, $R_{xx}(T)$ is linearly extrapolated to lower T (black line) and ΔR_{xx} is read off as indicated by the red arrow. **b**, Relative change of the longitudinal resistance $\Delta R_{xx}/R_{xx,0} = (\rho_{xx,0} - \rho_{xx})/\rho_{xx,0} = 1 - 1/(1 + (\tan\theta_{\mathrm{H}})^2)$ as function of the Hall angle (Eqn. 22, red line), together with the experimental data point determined from Supplementary Fig. S18a and Fig. 2c (black symbol).

The R_{xx} change is about 0.05% ($\approx$ 10 $\mu\Omega$), whereas R_{xy} changes by more than 5% ($\approx$ 140 $\mu\Omega$) between 0.9 K and 1.1 K. This effect can either arise from a small transverse misalignment of the longitudinal voltage contacts, or be an intrinsic imprint of R_{xy} on R_{xx}

through the Hall angle via

$$\rho_{xx} = \frac{\sigma_{xx}}{\sigma_{xx}^2 + \sigma_{xy}^2} = \frac{1}{\sigma_{xx}} \frac{1}{1 + (\tan\theta_{\rm H})^2} \ . \tag{22}$$

As the spontaneous Hall effect builds up with decreasing temperature, $\tan\theta_{\rm H}$ increases and, via Eqn. 22, ρ_{xx} drops. This effect was demonstrated for $Ce_3Bi_4Pd_3$ in the Supplementary Material of [4]. In Supplementary Fig. S18b we show that the theoretical expectation (Eqn. 22, red curve) and our experimental finding (black data point, from Supplementary Fig. S18a and Fig. 2c) agree reasonably well.

Supplementary Discussion 9: Nonlinearity in the DC transport response

The best-known characteristic of the spontaneous (nonlinear) Hall effect in time reversal symmetric but inversion symmetry broken systems as described in [35] is the Berry curvature dipole response, the first nonvanishing term in a perturbative treatment of the applied electric field. Its DC manifestation is a transverse voltage $V_{xy}^{\rm DC}$ in zero magnetic field that is square in the applied current I_x (whereas the longitudinal voltage $V_{xx}^{\rm DC}$ is linear in I_x). In the previously established Weyl-Kondo semimetal $Ce_3Bi_4Pd_3$, this Berry curvature dipole response was shown not to be the dominating contribution. Instead, a larger linear-in-I_x term of $V_{xy}^{\rm DC}$ was found and argued to result from a fully nonequilibrium situation that requires expansion around a finite-field setpoint and where time-reversal symmetry is broken thermodynamically [4]. More recently, this result was derived from Boltzmann transport theory [36]. In this section, we explain why the Berry curvature dipole response is further suppressed in $CeRu_4Sn_6$, to the point that it needs great care to be detectable at all.

In $Ce_3Bi_4Pd_3$, in the lowest temperature current-voltage isotherm (at 1.75 K, Supplementary Fig. S6A of [4]), an appreciable deviation from a linear $V_{xy}^{\rm DC}$ vs I_x characteristic could only be resolved at currents above 3 mA. This should not be misinterpreted as the I_x^2 term being absent at lower currents, but is simply because a quadratic function decreases more quickly at small currents than a linear function. Whereas a current of 3 mA does not overheat $Ce_3Bi_4Pd_3$ at 1.75 K, but below 1 K, the temperature range required to measure the spontaneous Hall effect in $CeRu_4Sn_6$, overheating occurs already at 2 mA (see below). Thus, extra efforts were needed, both in terms of measurement resolution and analysis, as described below.

High-resolution measurements of V_{xx} and V_{xy} as functions of the applied current I were performed at different set temperatures across the onset of the spontaneous Hall effect in $CeRu_4Sn_6$. For an unbiased analysis that captures any potential nonlinearity, each curve was fitted with a polynomial of degree 5, i.e., $V = V_0 + R^{(1)}I + R^{(2)}I^2 + R^{(3)}I^3 + R^{(4)}I^4 + R^{(5)}I^5$. As explained above, the (fully nonequilibrium) term $R^{(1)}I$ dominates. V_0 allows for a spurious voltage offset of the voltmeter. In Fig. S19, we plot $(V - V_0)/I = R^{(1)} + R^{(2)}I + R^{(3)}I^2 + R^{(4)}I^3 + R^{(5)}I^4$ for both V_{xy} (panel a) and V_{xx} (panel b) at temperatures above and below the onset of the spontaneous Hall effect. $R^{(1)}$ is the offset, and the (Berry curvature dipole) term $R^{(2)}$ (and $R^{(4)}$) appears as an asymmetry in the curves. For the xy channel, this asymmetry is much larger at low temperatures, but for the xx channel, it is small and essentially temperature independent.

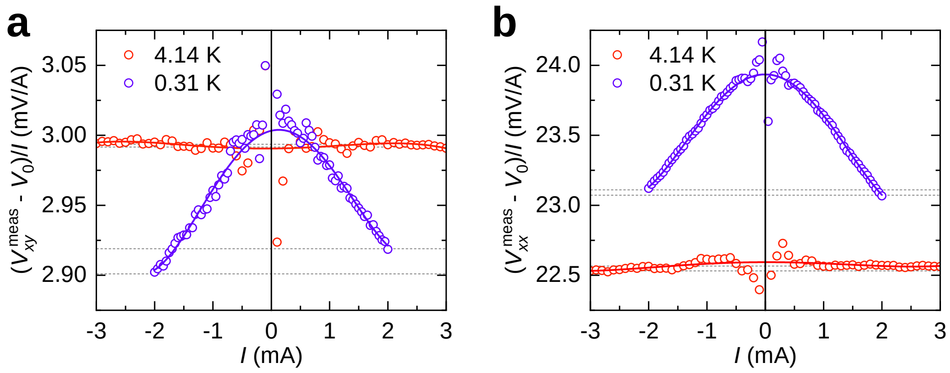

Fig. S19. **DC voltage-current characteristics of $CeRu_4Sn_6$.** Current dependence of $(V - V_0)/I$ for V_{xy} (**a**) and V_{xx} (**b**), above (red) and below (blue) the onset of the spontaneous Hall effect. The full lines are the polynomial fits, and the dashed horizontal lines help to discern the asymmetries in the curves, which are due to the Berry curvature dipole contribution of the spontaneous Hall effect (and an additional I^4 term, see text).

In addition, we plot all nonlinear coefficients $R^{(i)}$ in Fig. S20, as $R_{xy}^{(i)}$ and $\alpha R_{xx}^{(i)}$, where α is the misalignment correction factor. Note that the deviations from linearity are small and we are close to the resolution limit, as reflected by the large error bars. Nevertheless, some trends appear robust: (a) $R_{xy}^{(2)}$ increases at low temperatures, but $R_{xx}^{(2)}$ doesn't; (b) $R_{xy}^{(3)}$ and $\alpha R_{xx}^{(3)}$ deviate in a similar way from zero at low temperatures; (c,d) these trends are also reflected in the corresponding (even and odd-in-current) higher order terms. Identified as spontaneous Hall contributions can only be terms that appear in the xy channel but

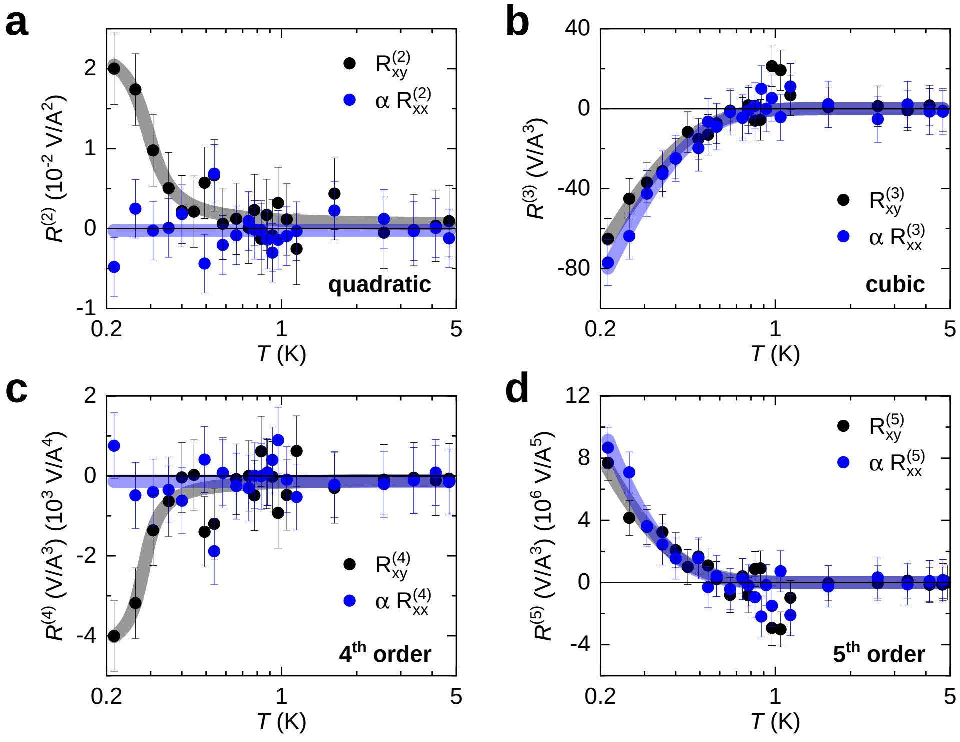

Fig. S20. **Nonlinearity in the DC transport response of $CeRu_4Sn_6$.** Temperature-dependent prefactors of the I^2 (**a**), I^3 (**b**), I^4 (**c**), and I^5 (**d**) terms of fits (see text) to the transverse (Hall) voltage V_{xy} (black) and the longitudinal voltage V_{xx} (blue), with the latter scaled by the same factor α as previously used for the contact misalignment correction. Data sets taken at similar T setpoints were averaged to increase the resolution. The error bars reflect the scattering of the data before averaging. Temperature corresponds to the average platform temperature during the current sweep; the average sample temperature is expected to be somewhat higher at low temperatures, due to slight overheating. Solid lines are guides to the eyes.

are absent in the xx channel, i.e., the (even) second- and fourth-order terms. The (odd) third- and fifth-order terms are attributed to overheating: at low temperatures, the current needed to resolve deviations from $V \sim R^{(1)}I$ heats the sample slightly, leading to a small decrease in resistance due to the negative temperature coefficient and thus the negative $R^{(3)}I^3$ contribution. The higher-order terms were included as a consistency check. That they behave as the corresponding lower-order terms reinforces the validity of our analysis.

In summary, with high-resolution experiments and an elaborate data analysis (that captures even minor nonlinearities and allows us to filter out small overheating effects), we were able to resolve the square-in-current Hall contribution expected within the Berry cur-

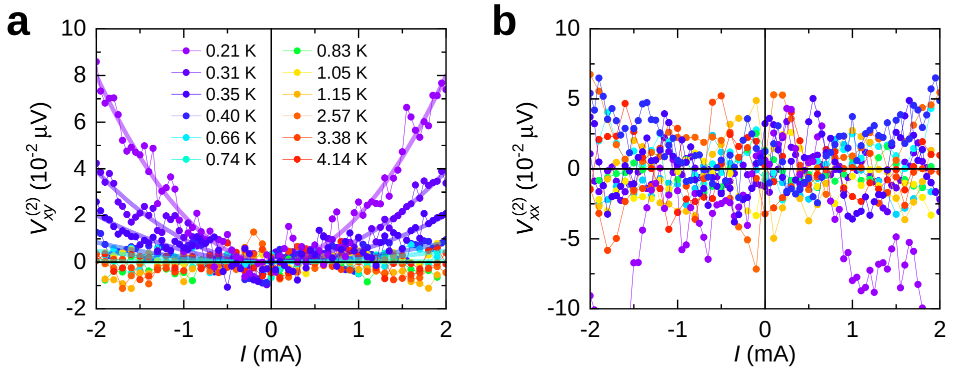

Fig. S21. **Quadratic contributions to the DC voltage-current curves of $\mathbf{CeRu_4Sn_6}$. a**, Current dependence of the $V_{xy}^{(2)}$ contribution, obtained by subtracting all other contributions of the polynomial fit from the as-measured data, for different temperatures. The shaded lines are the quadratic contribution of the fit. **b**, Same plot for V_{xx}, showing the absence of a systematic quadratic contribution.

vature dipole approximation (i.e., the finite low-temperature $R_{xy}^{(2)}$ term in Fig. S20a and the $V_{xy}^{(2)} \sim I_x^2$ dependence in Fig. S21a, together with the absence of these characteristics in the corresponding longitudinal channel). That the effect is small, even by comparison with $Ce_3Bi_4Pd_3$, can be attributed to two effects. (i) The onset temperature of the spontaneous Hall signal in $CeRu_4Sn_6$ is about 3 times lower than in $Ce_3Bi_4Pd_3$, thus allowing only for lower current densities without overheating the sample. (ii) $CeRu_4Sn_6$ is an emergent Weyl-Kondo semimetal that lacks well-defined quasiparticles, whereas $Ce_3Bi_4Pd_3$ is a "normal" Weyl-Kondo semimetal with well-defined Weyl-Kondo quasiparticles. The upper electric field limit of the Berry curvature dipole regime (defined by where the momentum space displacement of the Fermi surface induced by the applied electric field, k_E, is small compared to the distance of the Weyl node from the Fermi surface, k_{BC} [36]) is expected to be even lower because of the fuzziness of the Fermi surface and the broadness of the non-quasiparticle spectral functions.

Supplementary Discussion 10: Single phase nature of $CeRu_4Sn_6$ samples

Absence of inclusions in scanning electron microscopy. Scanning electron microscopy (SEM) is the method of choice to detect inclusions. Provided there is sufficient contrast (i.e. the inclusion and main phase have different compositions), inclusions with sizes above a few tens of nm can be readily resolved in our instrument. Energy-dispersive X-ray spectroscopy (EDX) can provide reliable information on the elemental composition of inclusions of sizes above about 1 μm in our instrument. Our SEM measurements on polished single crystals from the same batch as those used in the manuscript did not provide any indication of such inclusions (Fig. S22). The occasional black spots in the backscattered SEM images correspond to small holes in the crystal surface. EDX measurements taken at various points along the crystal all result in the same composition close to stoichiometry. It is thus unlikely that the observed spontaneous Hall signal relates to effects from foreign phases.

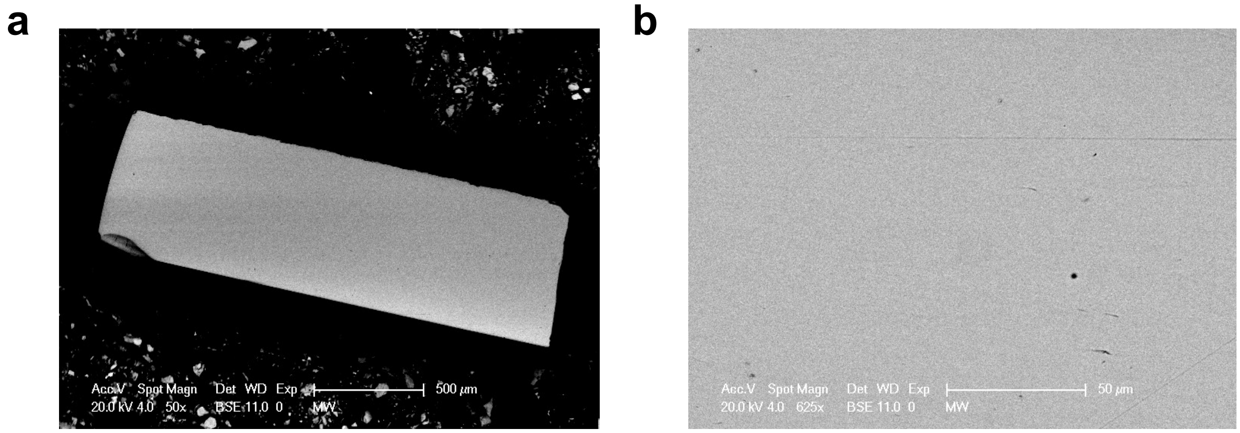

Fig. S22. **SEM study of a $CeRu_4Sn_6$ single crystal.** **a**, Backscattered SEM image of the polished single crystal surface showing no evidence for foreign phase inclusions. **b**, Representative close-up of a part of the sample, which shows a phase-pure crystal at higher resolution. The occasional dark spots correspond to small holes in the surface.

Absence of spontaneous Hall effect in $LaRu_4Sn_6$. To further support the intrinsic nature of the spontaneous Hall effect in $CeRu_4Sn_6$, we performed zero-field longitudinal and transverse voltage measurements on a single crystal of $LaRu_4Sn_6$ and analyzed the data in the same way as for $CeRu_4Sn_6$. Because $CeRu_4Sn_6$ and $LaRu_4Sn_6$ are grown under similar

conditions, any putative foreign phase that would form during the growth of $CeRu_4Sn_6$ might also form during the growth of $LaRu_4Sn_6$. The transverse signal in $LaRu_4Sn_6$ is completely featureless and essentially zero in the entire temperature range (Supplementary Fig. S23), further underpinning that the signals seen in $CeRu_4Sn_6$ and $CeRu_4(Sn_{5.8}Ge_{0.2})$ are spontaneous Hall signals in (emergent) Weyl-Kondo semimetals.

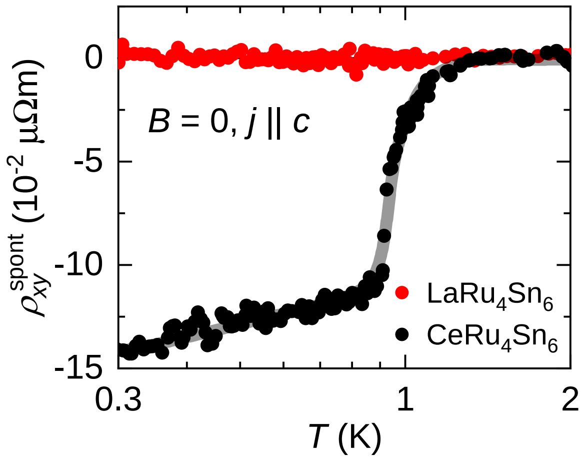

Fig. S23. **Absence of spontaneous Hall effect in $LaRu_4Sn_6$.** Zero-field Hall resistivity as a function of temperature for $CeRu_4Sn_6$ and $LaRu_4Sn_6$. The data for $LaRu_4Sn_6$ are featureless and essentially zero, demonstrating the absence of a spontaneous Hall effect as expected for a simple metal.

Superconductivity in hypothetical foreign phases. We also investigated whether the superconducting properties of any phase that could hypothetically form during the single crystal growth of $CeRu_4Sn_6$ could be confused with the effects we observed (and identified as spontaneous Hall signals). The $CeRu_4Sn_6$ single crystals were grown using the floating zone technique in a mirror furnace, with a self-flux consisting of 60% Ru_2Sn_3 and 40% Ru_3Sn_7. A phase that could potentially form under such conditions has the boundary compositions $CeRuSn_3$ and $Ce_3Ru_4Sn_{13}$. Furthermore, inclusions of elemental Ru were observed in some of the early growth attempts. For completeness, we also include the two flux components Ru_2Sn_3 and Ru_3Sn_7. Our literature search on superconducting properties of all these phases is summarized as follows:

- **$CeRuSn_3$:** In [37], resistivity, specific heat, and magnetic susceptibility were measured below 1 K. No signs of superconductivity were detected, in susceptibility measurements

down to 50 mK.

- **$CeRu_4Sn_{13}$:** In [38], the resistivity and specific heat of polycrystals were measured down to 0.4 K. No signs of superconductivity were observed. $LaRu_4Sn_{13}$, by contrast, was found to superconduct below 3.75 K.

- **Elemental Ru:** In bulk form, $T_c = (0.47 - 0.48)$ K and the critical field is 4.6 mT [39, 40]. T_c is almost pressure independent, with $\partial T_c/\partial p = (0 \pm 0.03) \cdot 10^{-5}$ K/atm [40]. For thin films, $T_c = (0.55 - 0.66)$ K and critical fields of $(0.85 - 3.83)$ T were reported [41].

- **Ru_2Sn_3:** In [42], a drop in $\rho(T)$ near 3.7 K is attributed to a minor contamination with Sn ($T_c = 3.75$ K). In [43], $\rho(T)$ decreases rather gradually below 3 K, but does not reach zero at the lowest measured temperature of 1 K. The authors discuss inclusions of Sn, oxides, an unknown Sn-based mixture, and partial superconductivity of an imperfect Ru_2Sn_3 crystal as possible origins.

- **Ru_3Sn_7:** Resistivity was measured down to 2 K, but no superconductivity was detected [44–46].

The documented superconducting characteristics of the first three materials are clearly distinct from the spontaneous Hall signal of $CeRu_4Sn_6$. Because for Ru_2Sn_3 and Ru_3Sn_7, literature gives no firm answer, we performed additional experiments: (i) we measured our flux rod, polycrystalline material composed of 60% Ru_2Sn_3 and 40% Ru_3Sn_7; (ii) we grew Ru_3Sn_7 single crystals and measured their resistivity. Both measurements reveal superconducting transitions below 1 K, but with characteristics that are quite different from those of the spontaneous and even-in-field Hall effect of $CeRu_4Sn_6$. Firstly, the spread of Weyl-Kondo semimetal onset temperatures T_H is small (6%, see Table I, Supplementary Discussion 2), whereas the superconducting transition temperatures of the two Ru_3Sn_7 crystals differ by 86%. Secondly, the temperature-magnetic field phase diagrams (Fig. S24) are distinct from the spontaneous and even-in-field Hall characteristics of $CeRu_4Sn_6$. In principle, filamentary superconductivity can exhibit enhanced upper critical fields, albeit only when the field is aligned with the filament, where orbital pair breaking is suppressed. We applied fields perpendicular to the sample plane, and the enhancement needed to match the $T_H(B)$ characteristics of the Weyl-Kondo semimetal phase in $CeRu_4Sn_6$ would work only for filaments

perpendicular to the sample plane. If these were long and reached up to the surface, we would have seen them in our SEM investigations.

Thus, none of the hypothetical foreign phases of $CeRu_4Sn_6$ have superconducting properties that could be confused with the observed spontaneous Hall signal.

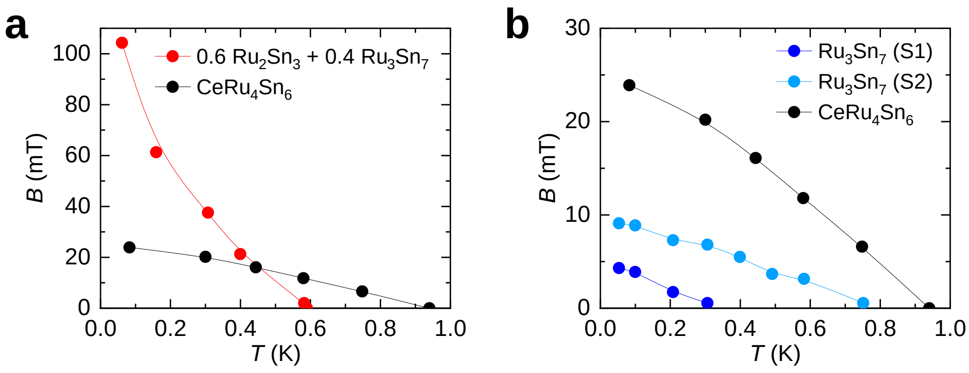

Fig. S24. **Superconductivity of hypothetical foreign phases vs Hall characteristics of $CeRu_4Sn_6$.** Phase diagram of critical field H as $B = \mu_0 H$ vs temperature T for the superconducting transitions of (**a**) the flux rod (composed of 60% Ru_2Sn_3 and 40% Ru_3Sn_7, red) and (**b**) two single crystals of Ru_3Sn_7 (blue), each compared to the onset temperatures of the spontaneous and even-in-field Hall effect of $CeRu_4Sn_6$ (black). The characteristics are distinct. Furthermore, the superconductivity is detected as usual in the (longitudinal) resistance, whereas the (spontaneous and even-in-field) Hall response in $CeRu_4Sn_6$ appears exclusively in the transverse ("Hall") channel.

Finite element simulations on a hypothetical superconducting inclusion. If a material contains superconducting inclusions, this typically manifests in smaller or larger jumps in the electrical resistivity, usually to smaller resistance values as the inclusion's transition to zero resistance short-circuits part of the voltage drop. As shown above, we have not found any indication of foreign phases in our $CeRu_4Sn_6$ single crystals. Nevertheless, one may ask whether a hypothetical superconducting inclusion could potentially produce effects reminiscent of the spontaneous Hall effect in $CeRu_4Sn_6$. In our experiments, the spontaneous Hall signal appears consistently in the transverse channel. A spurious signal on the longitudinal contacts could only be resolved in our highest resolution measurements; it is fully understood within the spontaneous Hall scenario (see Supplementary Discussion 8). Furthermore, the spontaneous Hall signal can be positive or negative (see Supplementary

Discussion 2, Sect. "Reproducibility").

We performed finite element simulations using COMSOL Multiphysics (AC/DC module, "Electric Currents" interface) to scrutinize this question. We find that, whereas peculiar shapes and arrangements of the inclusion can be found that produce a larger transverse than longitudinal voltage drop, such behavior is not generic; furthermore, the magnitude of the spontaneous Hall effect found in our experiments cannot be reproduced in the simulations. As an example, we present simulations on such a fine-tuned model (Fig. S25): a needle-shaped inclusion ($250 \times 5 \times 5\,\mu\text{m}^3$) placed $35\,\mu$m below the sample surface, such that it would be undetectable by SEM and EDX, and with a volume that is much smaller than the assumed sample volume, such that it would not be detected in specific heat measurements.

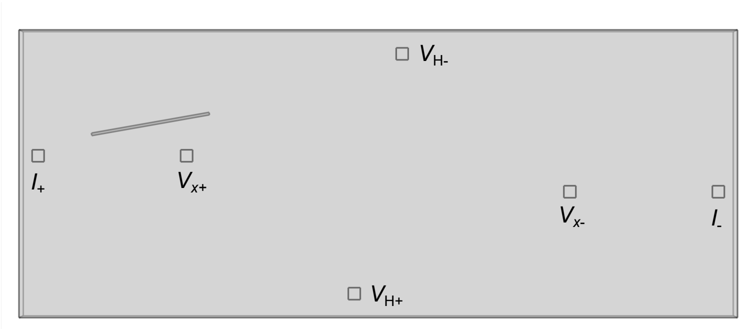

Fig. S25. **Fine-tuned model for finite element simulations.** Geometry of a hypothesized inclusion in an otherwise homogeneous sample (of dimensions $1500 \times 600 \times 100\,\mu\text{m}^3$), optimized for a larger change in the transverse than in the longitudinal resistance across the inclusion's superconducting transition.

In the simulations, we apply a current $I = 1\,\text{mA}$ between I_+ and I_- and measure the longitudinal resistance $R_{xx} = (V_{x+} - V_{x-})/I$ and the transverse resistance $R_{xy} = (V_{\text{H}+} - V_{\text{H}-})/I$. For simplicity, we assume the resistivity of the sample to be isotropic ($1.2\,\text{m}\Omega\text{cm}$ at $2\,\text{K}$) and that of the inclusion in its normal state to be the same. The superconducting state is modeled as $\rho^{\text{super}} = \rho^{\text{normal}}/10^{10}$.

To quantify the effect and compare it to our experimental results, we determine the ratio of transverse and longitudinal resistance changes across the inclusion's superconducting transition as

$$\left|\frac{\Delta R_{xy}}{\Delta R_{xx}}\right| = \left|\frac{R_{xy}^{\text{normal}} - R_{xy}^{\text{super}}}{R_{xx}^{\text{normal}} - R_{xx}^{\text{super}}}\right|$$

(Fig. S26). Repeating the simulation with slightly changed positions (x and y), rotation angles (θ) of the inclusion within the plane, and placements of the inclusion in different quadrants (see sketch in Fig. S26d) reveals the sensitivity of the effect to small geometrical changes, and thus the fine-tuned nature of the original model of Fig. S25. All simulated $|\Delta R_{xy}/\Delta R_{xx}|$ values are much smaller than the experimental result (red horizontal line), and the unusual effect of a larger transverse than longitudinal change is quickly suppressed (as points fall in the gray shaded area). The size of the effect is further reduced when (i) increasing the thickness of the sample (to match the experimental situation more closely) and (ii) adjusting the inclusion's normal state resistivity to that of a putative foreign phase (blue symbols in Fig. S26 are simulations with the inclusion's normal state resistivity set to the mean published resistivity of Ru_3Sn_7 at 2 K, $5.4 \cdot 10^{-4}$ mΩcm [44–46]).

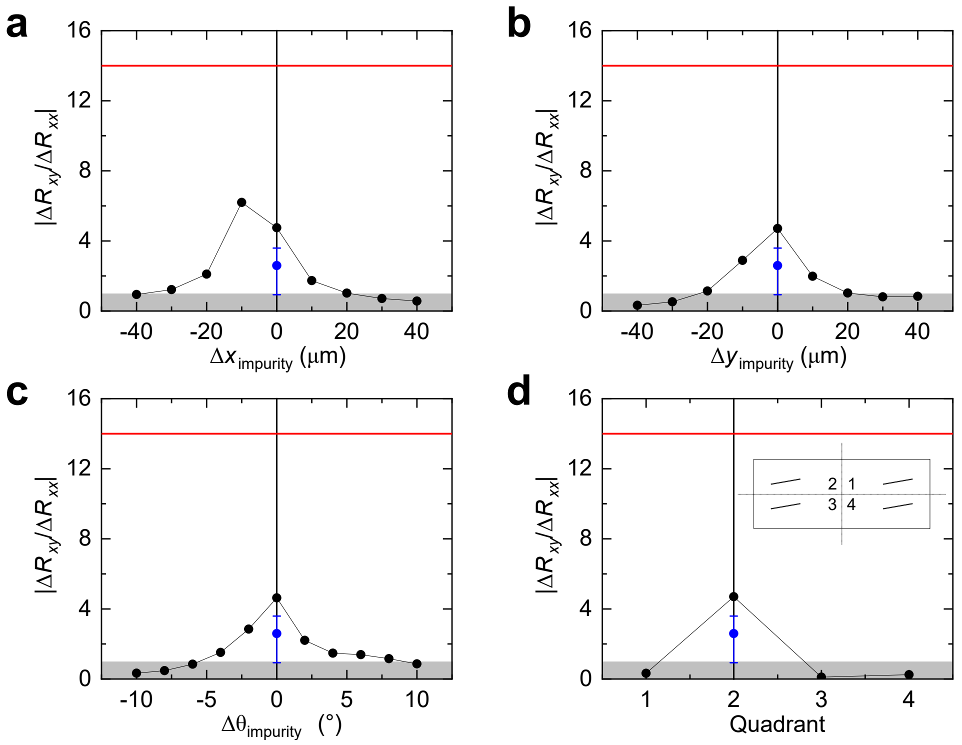

Fig. S26. **Geometry dependence of the studied model.** $|\Delta R_{xy}/\Delta R_{xx}|$ results of the simulation described in the text as function of the changes Δx (**a**), Δy (**b**), and $\Delta\Theta$ (**c**), and of different quadrant locations (**d**). The maximum effect is much smaller than in our experiments (red horizontal line). In the gray shaded area, the longitudinal signal is larger than the transverse one. The blue data point represents the result with Ru_3Sn_7 as inclusion (see text). The error bar spans the result for the largest and smallest published values.

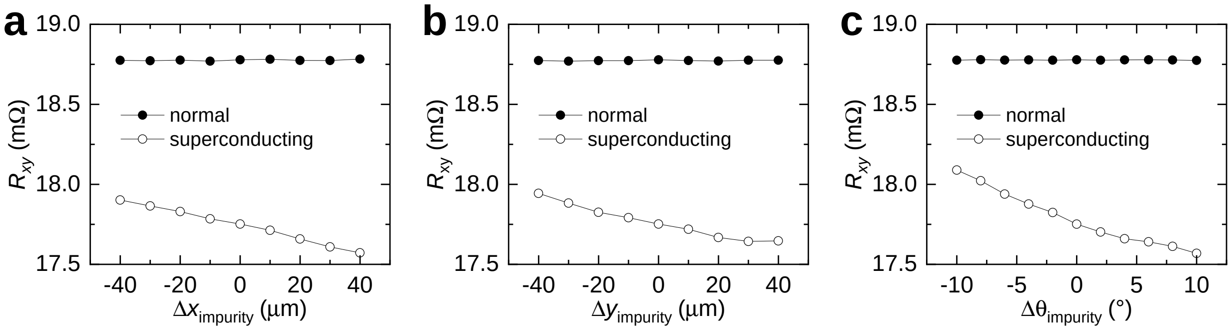

Fig. S27. **Sign of the resistance change.** R_{xy} (including the actual sign) in the normal and superconducting state of the hypothesized inclusion, for the model in Fig. S25 and small geometric variations thereof. As expected intuitively, the (positive valued) R_{xy} decreases significantly when the inclusion becomes superconducting.

Finally, to support our conclusions drawn from the positive sign of the spontaneous Hall signal in $CeRu_4Sn_{5.8}Ge_{0.2}$ (Supplementary Discussion 2, Sect. "Reproducibility"), we plot R_{xy}^{normal} and R_{xy}^{super} (including their actual signs) in Fig. S27. For all tested geometries, R_{xy}^{super} is smaller than R_{xy}^{normal}, confirming that the spontaneous Hall effect is clearly distinct from phenomena that can be created by a superconducting inclusion.

In summary, our finite element simulations support the assessment that the spontaneous Hall effect in $CeRu_4Sn_6$ is highly unlikely to result from a superconducting inclusion.

[1] Hayano, R. S., Uemura, Y. J., Imazato, J., Nishida, N., Yamazaki, T. & Kubo, R. Zero- and low-field spin relaxation studied by positive muons. *Phys. Rev. B* **20**, 850–859 (1979).

[2] K. Ishida, D. E. MacLaughlin, K. Okamoto, Y. Kawasaki, Y. Kitaoka, G. J. Nieuwenhuys, O. O. Bernal, A. Koda, W. Higemoto, R. Kadono, C. Geibel, and F. Steglich. μSR and low-temperature antiferromagnetism in the ordered non-Fermi-liquid compound $YbRh_2Si_2$. *Physica B* **329-333**, 589 (2003).

[3] Strydom, A. M., Hillier, A. D., Adroja, D. T., Paschen, S. & Steglich, F. Low-temperature muon spin relaxation measurements on $CeRu_4Sn_6$. *J. Magn. Magn. Mater.* **310**, 377 (2007).

[4] Dzsaber, S., Yan, X., Taupin, M., Eguchi, G., Prokofiev, A., Shiroka, T., Blaha, P., Rubel, O., Grefe, S. E., Lai, H.-H., Si, Q. & Paschen, S. Giant spontaneous Hall effect in a nonmagnetic Weyl-Kondo semimetal. *Proc. Natl. Acad. Sci. U.S.A.* **118**, e2013386118 (2021).

[5] Winkler, H., Lorenzer, K.-A., Prokofiev, A. & Paschen, S. Anisotropic electrical resistivity of the Kondo insulator $CeRu_4Sn_6$. *J. Phys. Conf. Series* **391**, 012077 (2012).

[6] Sullivan, P. F. & Seidel, G. Steady-state, ac-temperature calorimetry. *Phys. Rev.* **173**, 679–685 (1968).

[7] Hänel, J., Taupin, M., Ikeda, M., Martelli, V., Tomeš, P., Prokofiev, A. & Paschen, S. Thermal conductivity of the Kondo semiconductor $CeRu_4Sn_6$. *J. Phys. Conf. Series* **807**, 012013 (2017).

[8] Choi, E. S., Kang, H., Jo, Y. J. & Kang, W. Thermoelectric power measurement under hydrostatic pressure using a self-clamped pressure cell. *Rev. Sci. Inst.* **73**, 2999–3002 (2002).

[9] Wilhelm, H. & Jaccard, D. Alternating current calorimetry at very high pressure and low temperature. *J. Phys.: Condens. Matter* **14**, 10683–10687 (2002).

[10] Strydom, A. M., Guo, Z., Paschen, S., Viennois, R. & Steglich, F. Electronic properties of semiconducting $CeRu_4Sn_6$. *Physica B* **359–361**, 293–295 (2005).

[11] Dzsaber, S., Prochaska, L., Sidorenko, A., Eguchi, G., Svagera, R., Waas, M., Prokofiev, A., Si, Q. & Paschen, S. Kondo insulator to semimetal transformation tuned by spin-orbit coupling. *Phys. Rev. Lett.* **118**, 246601 (2017).

[12] Lai, H.-H., Grefe, S. E., Paschen, S. & Si, Q. Weyl-Kondo semimetal in heavy-fermion systems. *Proc. Natl. Acad. Sci. U.S.A.* **115**, 93 (2018).

[13] Paschen, S., Lühmann, T., Wirth, S., Gegenwart, P., Trovarelli, O., Geibel, C., Steglich, F., Coleman, P. & Si, Q. Hall-effect evolution across a heavy-fermion quantum critical point. *Nature* **432**, 881 (2004).

[14] Fuhrman, W. T., Sidorenko, A., Hänel, J., Winkler, H., Prokofiev, A., Rodriguez-Rivera, J. A., Qiu, Y., Blaha, P., Si, Q., Broholm, C. L. & Paschen, S. Pristine quantum criticality in a Kondo semimetal. *Sci. Adv.* **7**, eabf9134 (2021).

[15] Brüning, E. M., Brando, M., Baenitz, M., Bentien, A., Strydom, A. M., Walstedt, R. E. & Steglich, F. Low-temperature properties of $CeRu_4Sn_6$ from NMR and specific heat measurements: Heavy fermions emerging from a Kondo-insulating state. *Phys. Rev. B* **82**, 125115 (2010).

[16] Childs, W. J. & Goodman, L. S. Magnetic hyperfine structure of the $^3P_1$ and $^3P_2$ metastable states of $Sn^{115,117,119}$. *Phys. Rev.* **137**, A35–A42 (1965).

[17] Büttgenbach, S., Dicke, R., Gebauer, H. & Herschel, M. Hyperfine structure and nuclear moments of ^{99}Ru and ^{101}Ru. *Z. Phys. A* **280**, 217–226 (1977).

[18] Zhang, J., Zhang, S., Chen, Z., Lv, M., Zhao, H., feng Yang, Y., Chen, G. & Sun, P. Pressure effect in the Kondo semimetal $CeRu_4Sn_6$ with nontrivial topology. *Chin. Phys. B* **27**, 097103 (2018).

[19] Cooley, J. C., Aronson, M. C. & Canfield, P. C. High pressures and the Kondo gap in $Ce_3Bi_4Pt_3$. *Phys. Rev. B* **55**, 7533–7538 (1997).

[20] Breindel, A. J., Deng, Y., Moir, C. M., Fang, Y., Ran, S., Lou, H., Li, S., Zeng, Q., Shu, L., Wolowiec, C. T., Schuller, I. K., Rosa, P. F. S., Fisk, Z., Singleton, J. & Maple, M. B. Probing FeSi, a *d*-electron topological Kondo insulator candidate, with magnetic field, pressure, and microwaves. *Proc. Natl. Acad. Sci. U.S.A.* **120**, e2216367120 (2023).

[21] Guritanu, V., Wissgott, P., Weig, T., Winkler, H., Sichelschmidt, J., Scheffler, M., Prokofiev, A., Kimura, S., Iizuka, T., Strydom, A. M., Dressel, M., Steglich, F., Held, K. & Paschen, S. Anisotropic optical conductivity of the putative Kondo insulator $CeRu_4Sn_6$. *Phys. Rev. B* **87**, 115129 (2013).

[22] Dzsaber, S., Zocco, D. A., McCollam, A., Weickert, F., McDonald, R., Taupin, M., Yan, X., Prokofiev, A., Tang, L. M. K., Vlaar, B., Winter, L. E., Jaime, M., Si, Q. & Paschen, S. Control of electronic topology in a strongly correlated electron system. *Nat. Commun.* **13**, 5729 (2022).

[23] Hu, H., Chen, L., Setty, C., Garcia-Diez, M., Grefe, S. E., Prokofiev, A., Kirchner, S., Vergniory, M. G., Paschen, S., Cano, J. & Si, Q. Topological semimetals without quasiparticles, *arXiv:2110.06182* (2021).

[24] Grefe, S. E., Lai, H.-H., Paschen, S. & Si, Q. Weyl-Kondo semimetals in nonsymmorphic systems. *Phys. Rev. B* **101**, 075138 (2020).

[25] Hu, H., Chen, L. & Si, Q. Extended dynamical mean field theory for correlated electron models, *arXiv:2210.14197* (2022).

[26] Vidberg, H. J. & Serene, J. W. Solving the Eliashberg equations by means of N-point Padé approximants. *J. Low Temp. Phys.* **29**, 179–192 (1977).

[27] Checkelsky, J. G., Bernevig, B. A., Coleman, P., Si, Q. & Paschen, S. Flat bands, strange metals, and the Kondo effect. *Nat. Rev. Mater.* **9**, 509–526 (2024).

[28] Xu, Y., Yue, C., Weng, H. & Dai, X. Heavy Weyl fermion state in $CeRu_4Sn_6$. *Phys. Rev. X* **7**, 011027 (2017).

[29] Chen, L., Lowder, D. T., Bakali, E., Andrews, A. M., Schrenk, W., Waas, M., Svagera, R., Eguchi, G., Prochaska, L., Wang, Y., Setty, C., Sur, S., Si, Q., Paschen, S. & Natelson, D. Shot noise in a strange metal. *Science* **382**, 907–911 (2023).

[30] Braß, M., Tomczak, J. M. & Held, K. Weyl nodes in $Ce_3Bi_4Pd_3$ revealed by dynamical mean-field theory. *Phys. Rev. Res.* **6**, 033227 (2024).

[31] Desranges, H.-U. & Schotte, K. D. Specific heat of the Kondo model. *Phys. Lett. A* **91**, 240 (1982).

[32] Hundley, M. F., Canfield, P. C., Thompson, J. D., Fisk, Z. & Lawrence, J. M. Hybridization gap in $Ce_3Bi_4Pt_3$. *Phys. Rev. B* **42**, 6842 (1990).

[33] Aeppli, G. & Fisk, Z. Kondo insulators. *Comments Condens. Matter Phys.* **16**, 155 (1992).

[34] Kushwaha, S. K., Chan, M. K., Park, J., Thomas, S. M., Bauer, E. D., Thompson, J. D., Ronning, F., Rosa, P. F. S. & Harrison, N. Magnetic field-tuned Fermi liquid in a Kondo insulator. *Nat. Commun.* **10**, 5487 (2019).

[35] Sodemann, I. & Fu, L. Quantum nonlinear Hall effect induced by Berry curvature dipole in time-reversal invariant materials. *Phys. Rev. Lett.* **115**, 216806 (2015).

[36] Sur, S., Chen, L., Wang, Y., Setty, C., Paschen, S. & Si, Q. Fully nonequilibrium Hall response from Berry curvature, *arXiv:2411.16675* (2024).

[37] Takayanagi, S., Fukuhara, T., Sato, H., Wada, N. & Yamada, Y. Heavy fermion behaviour in

$CeRuSn_3$ compound. *Physica B: Condens. Matter* **165-166**, 447–448 (1990).

[38] Ślebarski, A., Fijałkowski, M., Goraus, J., Kalinowski, L. & Witas, P. Crystal electric field and the ground state properties of heavy fermion $Ce_3Ru_4Sn_{13}$. *J. Alloys Compd.* **615**, 921–928 (2014).

[39] Goodman, B. B. Two new superconducting elements. *Nature* **167**, 111 (1951).

[40] Bucher, E., Müller, J., Olsen, J. & Palmy, C. Superconductivity of osmium and ruthenium under pressure. *Cryogenics* **5**, 283–284 (1965).

[41] Langa Jr., B., Henry, B., Lainez, I., Haight, R. & Sardashti, K. A study of superconducting behavior in ruthenium thin films. *APL Mater.* **13**, 061114 (2025).

[42] Shiomi, Y. & Saitoh, E. Linear magnetoresistance in a topological insulator Ru_2Sn_3. *AIP Adv.* **7**, 035011 (2017).

[43] Wu, B., Barrena, V., Mompeán, F., García-Hernández, M., Suderow, H. & Guillamón, I. Linear nonsaturating magnetoresistance in the Nowotny chimney ladder compound Ru_2Sn_3. *Phys. Rev. B* **101**, 205123 (2020).

[44] Chakoumakos, B. C. & Mandrus, D. Ru_3Sn_7 with the Ir_3Ge_7 structure-type. *J. Alloys Compd.* **281**, 157–159 (1998).

[45] Tran, V. & Miiller, W. Ru_3Sn_7: Phonon reference for superconducting Mo_3Sb_7. *Acta Phys. Pol. A* **115**, 83–85 (2009).

[46] Ji, X., Zhou, X., Zhu, S., Ma, F., Li, G. & Wu, W. Dirac point in the charge compensated single-crystal Ru_3Sn_7. *Materials* **18**, 4044 (2025).
